# Author Correction: Single-component multilayered self-assembling nanoparticles presenting rationally designed glycoprotein trimers as Ebola virus vaccines

**DOI:** 10.1038/s41467-022-34108-9

**Published:** 2022-10-25

**Authors:** Linling He, Anshul Chaudhary, Xiaohe Lin, Cindy Sou, Tanwee Alkutkar, Sonu Kumar, Timothy Ngo, Ezra Kosviner, Gabriel Ozorowski, Robyn L. Stanfield, Andrew B. Ward, Ian A. Wilson, Jiang Zhu

**Affiliations:** 1 Department of Integrative Structural and Computational Biology, The Scripps Research Institute, La Jolla, CA USA; 2 Skaggs Institute for Chemical Biology, The Scripps Research Institute, La Jolla, CA USA; 3 Department of Immunology and Microbiology, The Scripps Research Institute, La Jolla, CA USA

**Keywords:** Humoral immunity, Protein vaccines, X-ray crystallography

Correction to: *Nature Communications* 10.1038/s41467-021-22867-w, published online 11 May 2021

The original version of this Article contained errors in Fig. 2a and the corresponding Results. In ‘Effect of the HR1C bend on EBOV GP metastability’ in Results, it incorrectly read ‘…forms a 21-nm triangular interior at around two-thirds of the height of the chalice that spans 94-Å wide at the rim…’. The correct version states ‘21-Å’ in place of ‘21-nm’. Fig. 2a incorrectly used ‘nm’ as unit, rather than the correct ‘Å’.

This has been corrected in both the PDF and HTML versions of the Article.

