# Supplementary material for: Single-component multilayered self-assembling nanoparticles presenting rationally designed glycoprotein trimers as Ebola virus vaccines
Source: Nat Commun. 2021 May 11;12:2633. doi: 10.1038/s41467-021-22867-w (PMC8113551; doi:10.1038/s41467-021-22867-w)
Supplement: Supplementary file 1 — Supplementary Information [file 41467_2021_22867_MOESM1_ESM.pdf]

Fig. S1

a **b** **c**

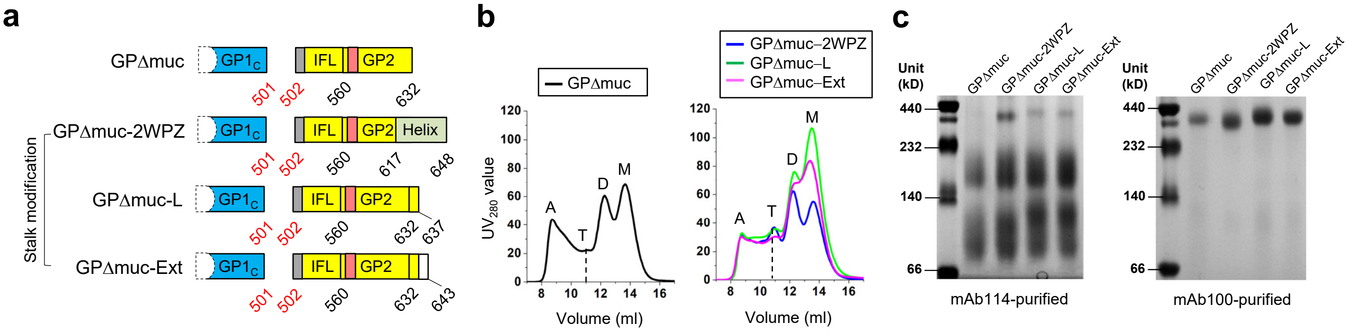

d **Antigenic profiling of four mAb100/SEC-purified, stalk-modified EBOV GP/GPΔmuc-foldon trimers**

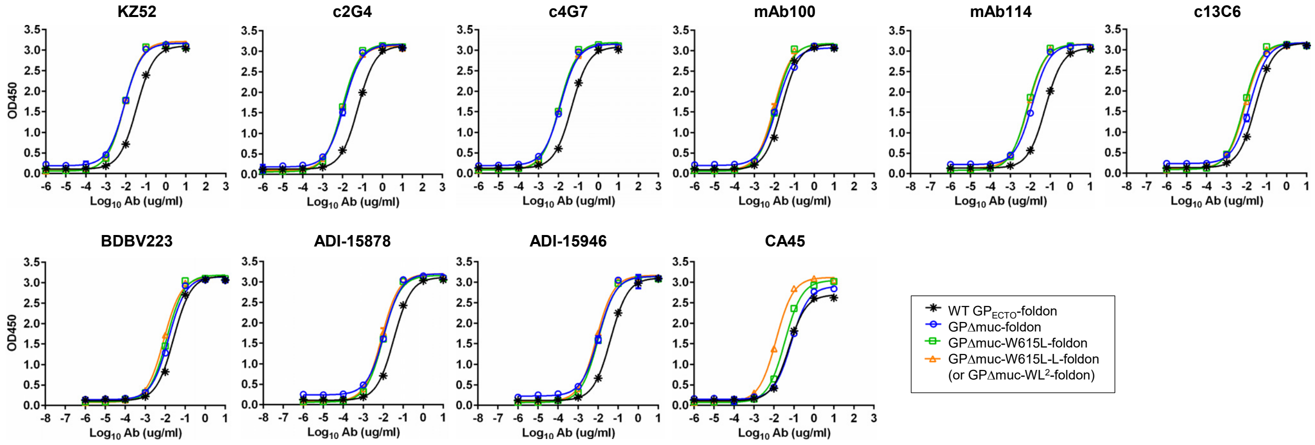

e

EC_{50} values (μg/ml) of four EBOV GP/GPΔmuc trimers binding to 10 antibodies [a].

	KZ52	c2G4	c4G7	mAb100	mAb114	c13C6	BDBV223	ADI-15878	ADI-15946	CA45
WT GP_{ECTO}-foldon	0.035	0.059	0.045	0.024	0.056	0.028	0.026	0.034	0.042	0.056
GPΔmuc-foldon	0.009	0.012	0.013	0.013	0.012	0.015	0.015	0.011	0.010	0.073
GPΔmuc-W615L-foldon	0.008	0.009	0.011	0.011	0.007	0.007	0.012	0.010	0.010	0.035
GPΔmuc-WL²-foldon	0.008	0.010	0.011	0.010	0.008	0.008	0.008	0.008	0.008	0.013

[a] EC_{50} values were calculated from the besting fitting in GraphPad Prism v8.4.3.

f **Antigenic profiling of an mAb100/SEC-purified, stalk/$HR1_C$-modified EBOV GPΔmuc-foldon trimer**

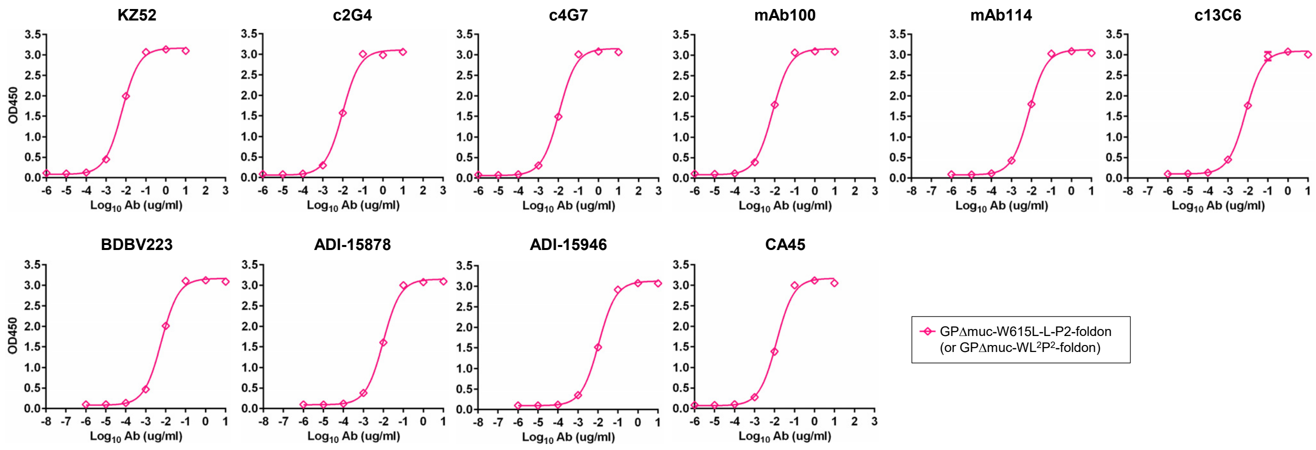

g

EC_{50} values (μg/ml) of a stalk/$HR1_C$-modified EBOV GPΔmuc trimer binding to 10 antibodies [a].

	KZ52	c2G4	c4G7	mAb100	mAb114	c13C6	BDBV223	ADI-15878	ADI-15946	CA45
GPΔmuc-WL²P²-foldon	0.006	0.010	0.011	0.008	0.007	0.008	0.006	0.010	0.011	0.012

[a] EC_{50} values were calculated from the besting fitting in GraphPad Prism v8.4.3.

Fig. S1

h

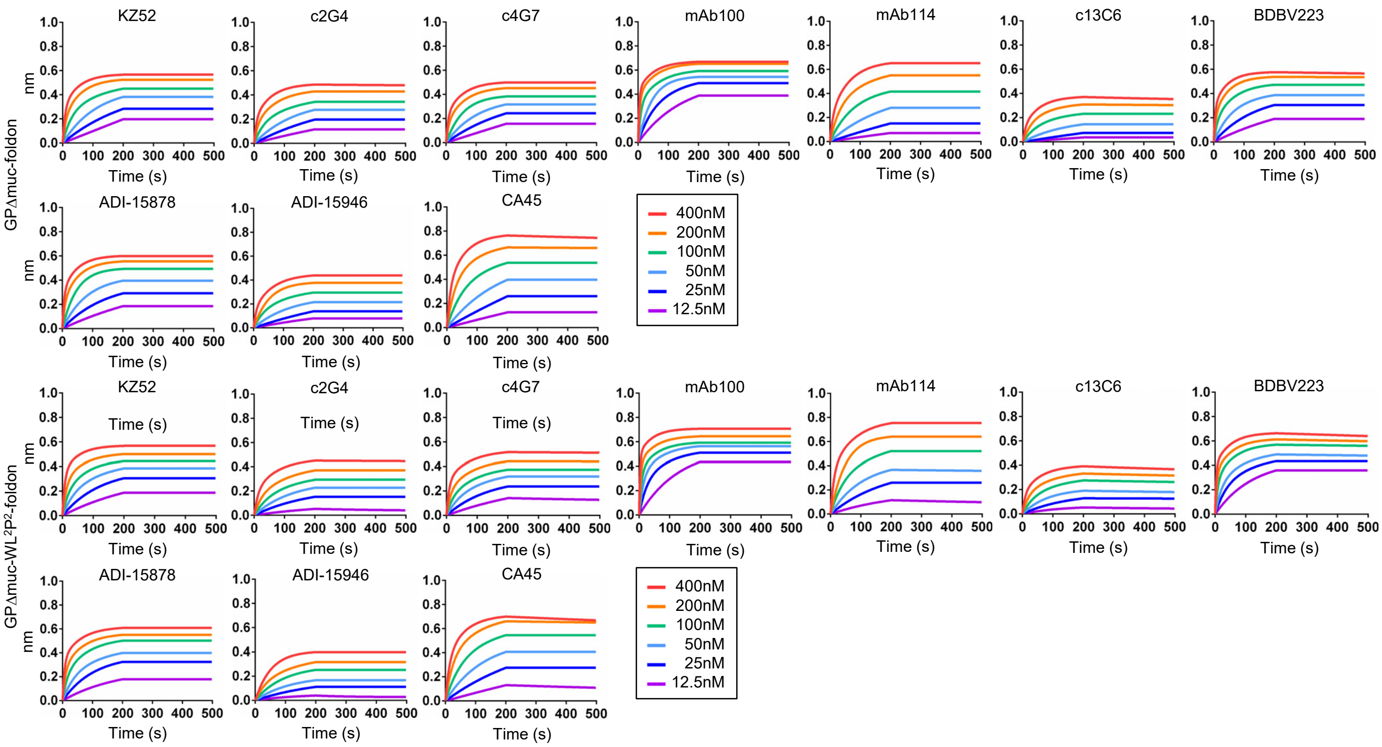

Fig. S1. Design, screening, and antigenic characterization of EBOV GPΔmuc trimers with modified stalk and HR1$_C$. (**a**) Schematic representation of mucin-deleted GP (GPΔmuc) and three stalk designs (GPΔmuc-2WPZ, GPΔmuc-L, and GPΔmuc-Ext). In the GPΔmuc-2WPZ construct design, in addition to replacing aa 617-632 with the coiled-coil region (aa 3-33) of 2WPZ, a double mutation (P612G/W615F) was introduced to reduce structural strain within the mutated stalk. (**b**) SEC profiles of four GPΔmuc constructs obtained from a Superdex 200 10/300 column following transient expression in 250 ml HEK293 F cells and mAb114 purification. Left: the SEC curve of GPΔmuc shown in black line; Right: three stalk designs shown in blue, green, and magenta lines for GPΔmuc-2WPZ, -L, and -Ext, respectively. (**c**) BN-PAGE of WT GPΔmuc and three stalk designs (GPΔmuc-2WPZ, -L, and -Ext) purified by mAb114 (left) and mAb110 (right) columns. (**d**) ELISA curves of four mAb100/SEC-purified, stalk-modified EBOV GP/GPΔmuc-foldon trimers binding to 10 antibodies. (**e**) Summary of EC$_{50}$ values (μg/ml) of EBOV GP/GPΔmuc-foldon trimers binding to 10 antibodies. **(f)** ELISA curves of an mAb100/SEC-purified, stalk/HR1$_C$-modified EBOV GPΔmuc-foldon trimer binding to 10 antibodies. (**g**) Summary of EC$_{50}$ values (μg/ml) of a stalk/HR1$_C$-modified EBOV GPΔmuc-foldon trimer binding to 10 antibodies. In (e) and (g), EC$_{50}$ values were calculated for all ELISA data in Prism 8.4.3. (**h**) Antigenic evaluation of GPΔMuc-foldon and GPΔmuc-WL^{2}P^2-foldon trimers using BLI and 10 representative antibodies. Sensorgrams were obtained from an Octet RED96 instrument using six concentrations (400-12.5 nM by twofold dilution) and AHC biosensors (see Methods). Source data are provided as a Source Data file.

Fig. S2

a

Inter-protomer disulfide bond (SS) mutations [a]

#	Res1	Res2	$d_{C\beta}$	Comments
**SS1**	TYR534	PHE153	3.82	IFL-head
**SS2**	ALA538	GLY91	3.99	IFL-head
**SS3**	THR520	ALA575	4.01	IFL-NHR
—	TRP615	ILE603	4.14	Hydrophobic cluster
**SS4**	ILE532	GLY157	4.20	IFL-head
**SS5**	ASP522	ALA575	4.41	IFL-NHR
	GLU611	HIS602	4.59	SS cysteine
**SS6**	LYS56	GLY599	4.60	Stalk (HR2)
—	CYS609	THR600	4.62	SS cysteine
	TRP531	GLN567	4.73	$d_{C\beta}$ > **cutoff**

[a] C_β-C_β distance of 4.72 Å as the indicator of S-S bond formation

b

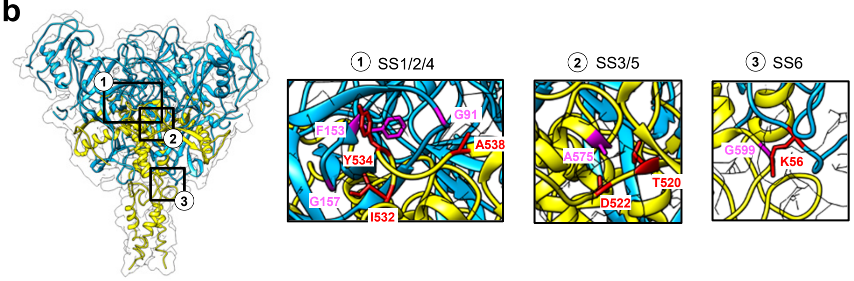

c

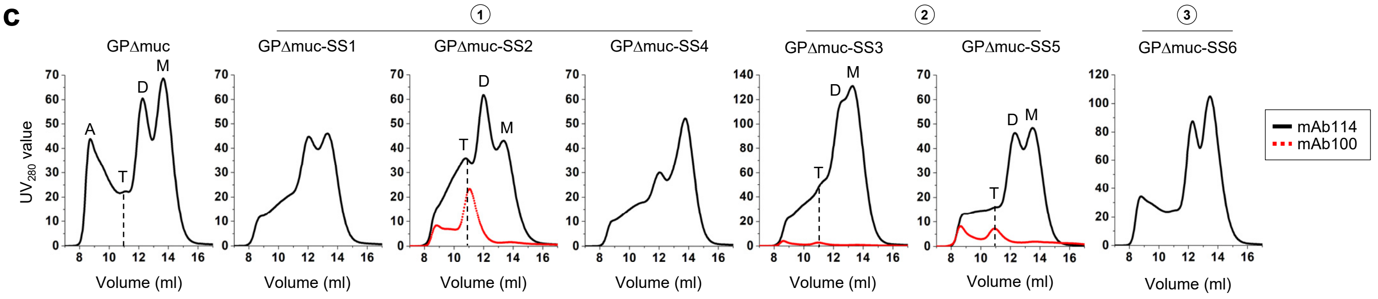

d

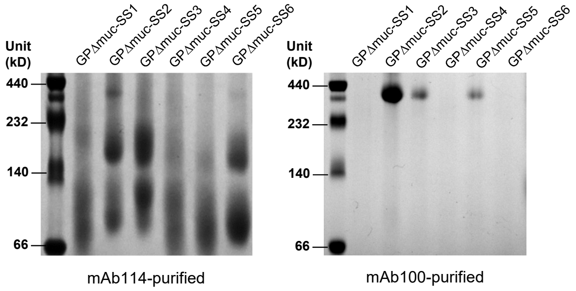

e

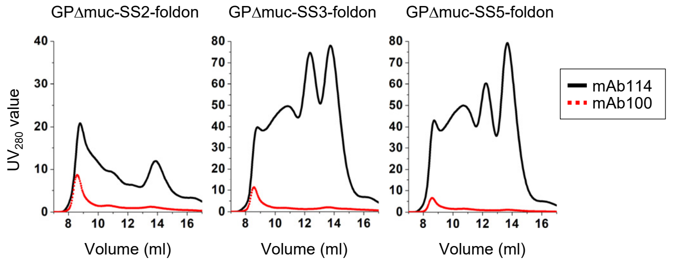

f **Antigenic profiling of mAb100/SEC-purified GPΔmuc-SS2 trimer (without foldon)**

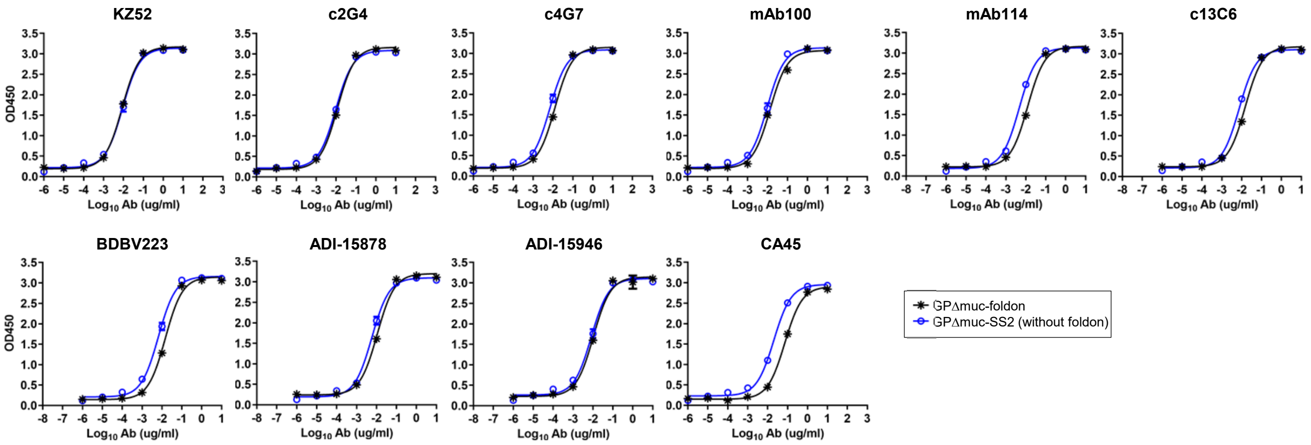

g

EC_{50} values (μg/ml) of EBOV GPΔmuc-SS2 trimer binding to 10 antibodies [a].

	KZ52	c2G4	c4G7	mAb100	mAb114	c13C6	BDBV223	ADI-15878	ADI-15946	CA45
GPΔmuc-foldon	0.009	0.012	0.013	0.013	0.012	0.015	0.015	0.011	0.010	0.073
GPΔmuc-SS2	0.009	0.010	0.007	0.010	0.005	0.007	0.007	0.006	0.008	0.020

[a] EC_{50} values were calculated from the besting fitting in GraphPad Prism v8.4.3. ELISA data for the GPΔmuc-foldon trimer is included for comparison.

Fig. S2. Design and characterization of EBOV GPΔmuc trimers with inter-protomer disulfide bonds. (**a**) Inter-protomer disulfide bond (SS) mutations identified using the atomic structure of a WT GPΔmuc trimer (PDB: 5JQ3) with residue numbers, C_β-C_β distance, and location listed as the key parameters for selection. (**b**) Structure and molecular surface of EBOV GPmMuc trimer (PDB: 5JQ3) in transparent molecular surface with the three SS classes labeled according to their locations. Zoomed-in views of the three SS classes, SS1/2/4, SS3/5, and SS6, are shown on the right. (**c**) SEC profiles of mAb114-purified GPΔmuc and six GPΔmuc-SS variants from a Superdex 200 10/300 column. SEC profiles of mAb100-purified SS2, SS3, and SS5 are shown in the red dotted line. Trimer (T), dimer (D), and monomer (M) peaks are labeled on the SEC profiles for SS2, SS3 and SS5, with the trimer peak marked with a black dashed line. (**d**) BN-PAGE of six GPΔmuc-SS variants after purification using an mAb114 column (left) and an mAb100 column (right). (**e**) SEC profiles of GPΔmuc-SS2/3/5-foldon constructs after purification using an mAb114 column (in the black line) and an mAb100 column (in the red dotted line) purification. No trimer peak was observed for any of the three GPΔmuc-SS-foldon constructs. (**f**) ELISA curves of the mAb100/SEC-purified GPΔmuc-SS2 trimer binding to 10 representative antibodies. (**g**) Summary of EC_{50} values (μg/ml) of the mAb100/SEC-purified GPΔmuc-SS2 trimer binding to 10 representative antibodies. In (f) and (g), ELISA data for the GPΔmuc-foldon trimer are included for comparison. EC_{50} values were calculated for all ELISA data in Prism 8.4.3. Source data are provided as a Source Data file.

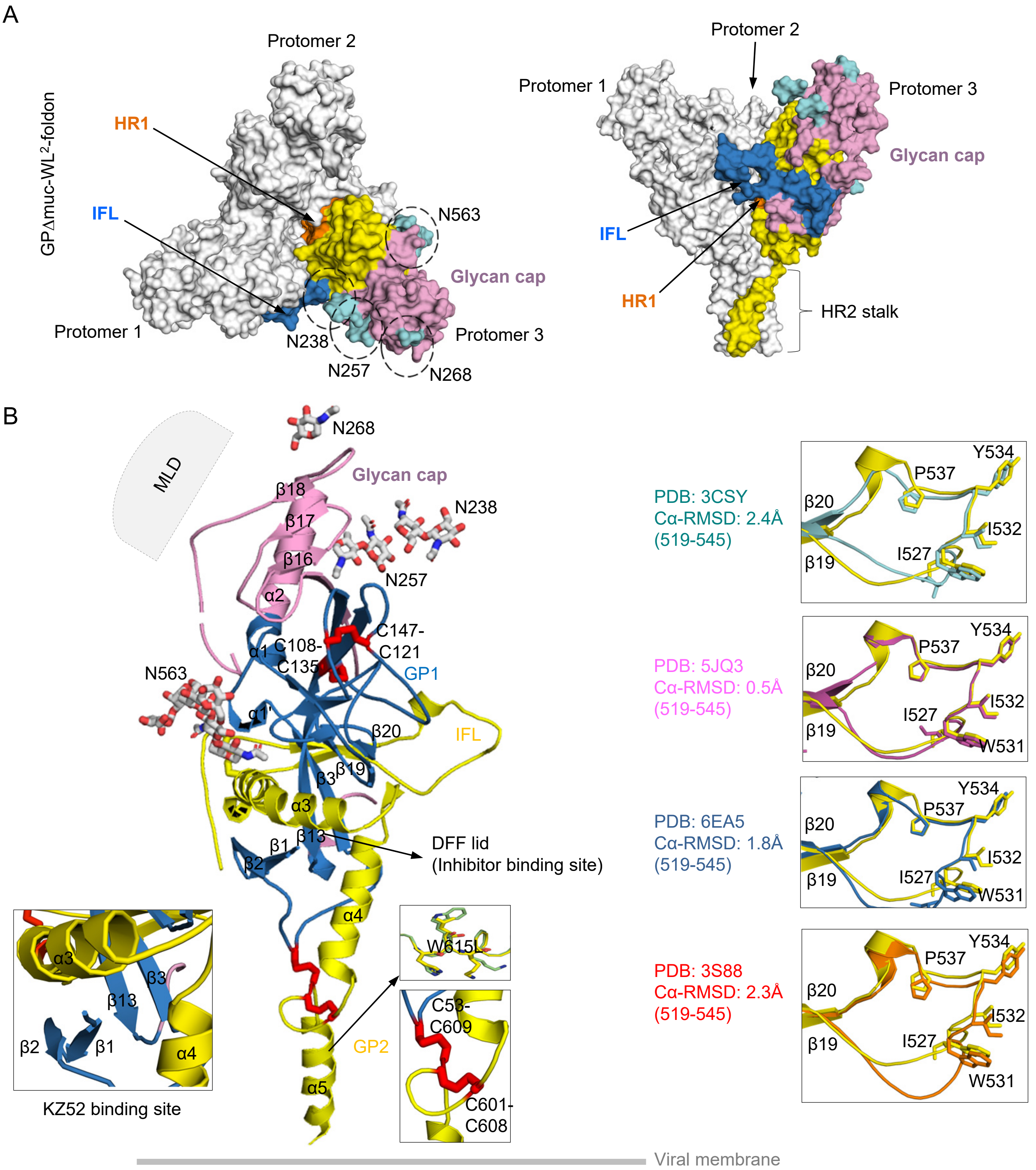

Fig. S3. Crystal structure of GPΔmuc-WL²-foldon at 2.3 Å resolution. (**a**) Molecular surface representation of GPΔmuc-WL²-foldon trimer structure (Left: top view; Right: side view). Protomers 1 and 2 are shown in light grey and protomer 3 is colored as follows: HR1 (orange), IFL(blue), glycan cap (pink), and HR2 (yellow). *N*-linked glycans at N238, N257, N268 and N563 are highlighted in circles and colored in cyan. (**b**) Ribbon representation of a GPΔmuc-WL²-foldon protomer with the same color scheme as in (a). Glycans and disulfide bonds are shown as color-coded (by atom type) and red sticks, respectively. The mucin-like domain (MLD) is shown as a gray oval. Binding sites for KZ52 and small-molecule inhibitors are indicated. The W615L mutation and two disulfide bonds (C53-C609 and C601-C608) are shown as insets. Superposed IFLs (R519-E545) from WL² and known structures (PDB IDs: 3CSY, 5JQ3, 6EA5, and 3S88) are shown as insets (right), with Cα RMSDs of 1.8-2.4Å.

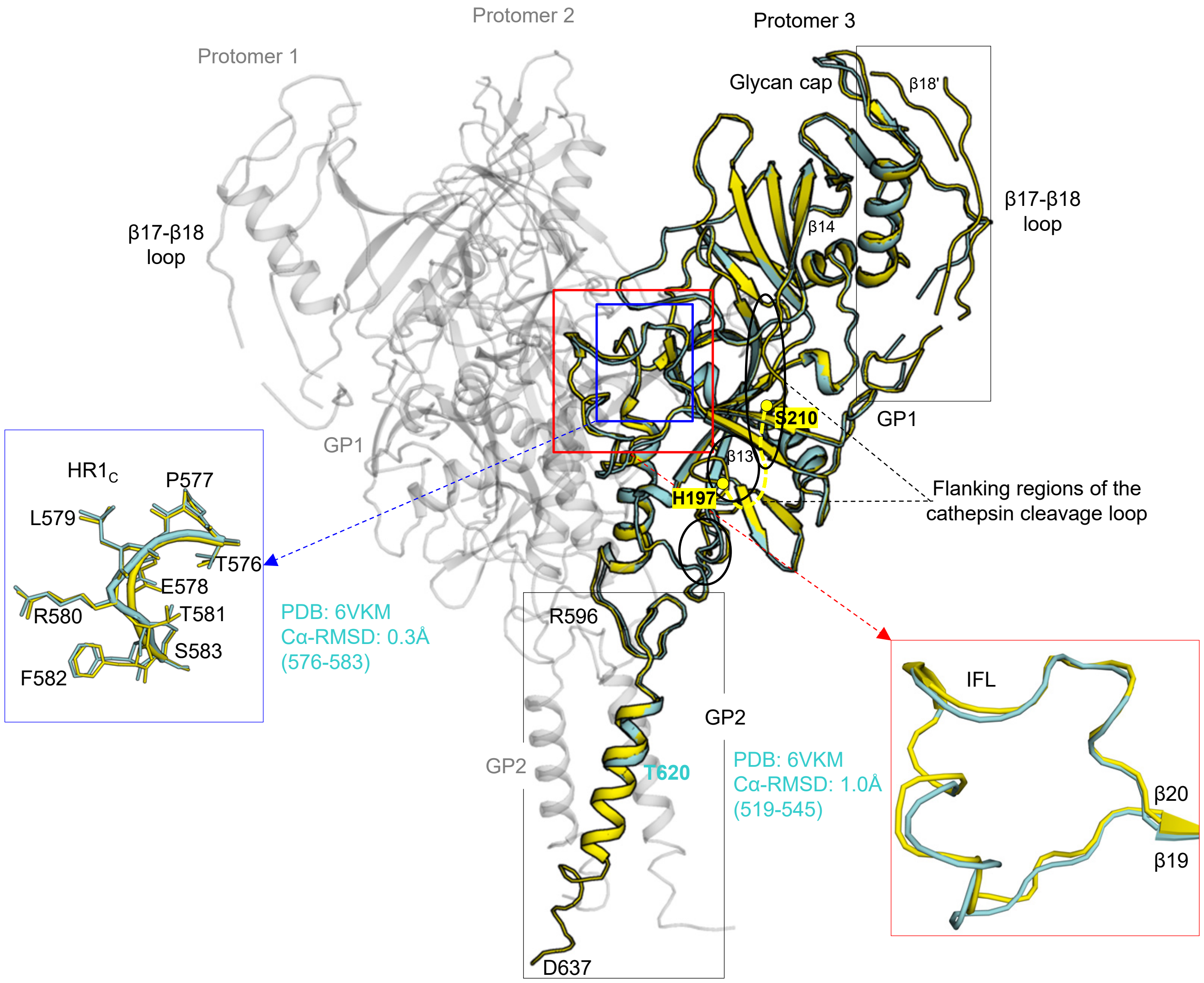

Fig. S4. Comparison of the Mayinga GPΔmuc-WL²P²-foldon structure and the previously reported Makona GPΔmuc structure containing a T577P/R588E double mutation. Ribbon representation of the GPΔmuc-WL²P²-foldon trimer structure is shown, with protomers 1/2 and 3 colored in light grey and yellow, respectively. The structure of a Makona GPΔmuc protomer (PDB: 6VKM) is superimposed onto protomer 3 and shown in cyan. Superimposed $HR1_C$ regions and internal fusion loops (IFL) from the two structures are shown in the bottom left and right insets, respectively. The flanking regions of the cathepsin cleavage loop (between β13 and β14, aa 190-210) are indicated in black ovals. H197 and S210 are labeled. The unstructured cathepsin cleavage loop between H197 and S210 is indicated as a yellow dashed line.

Fig. S5

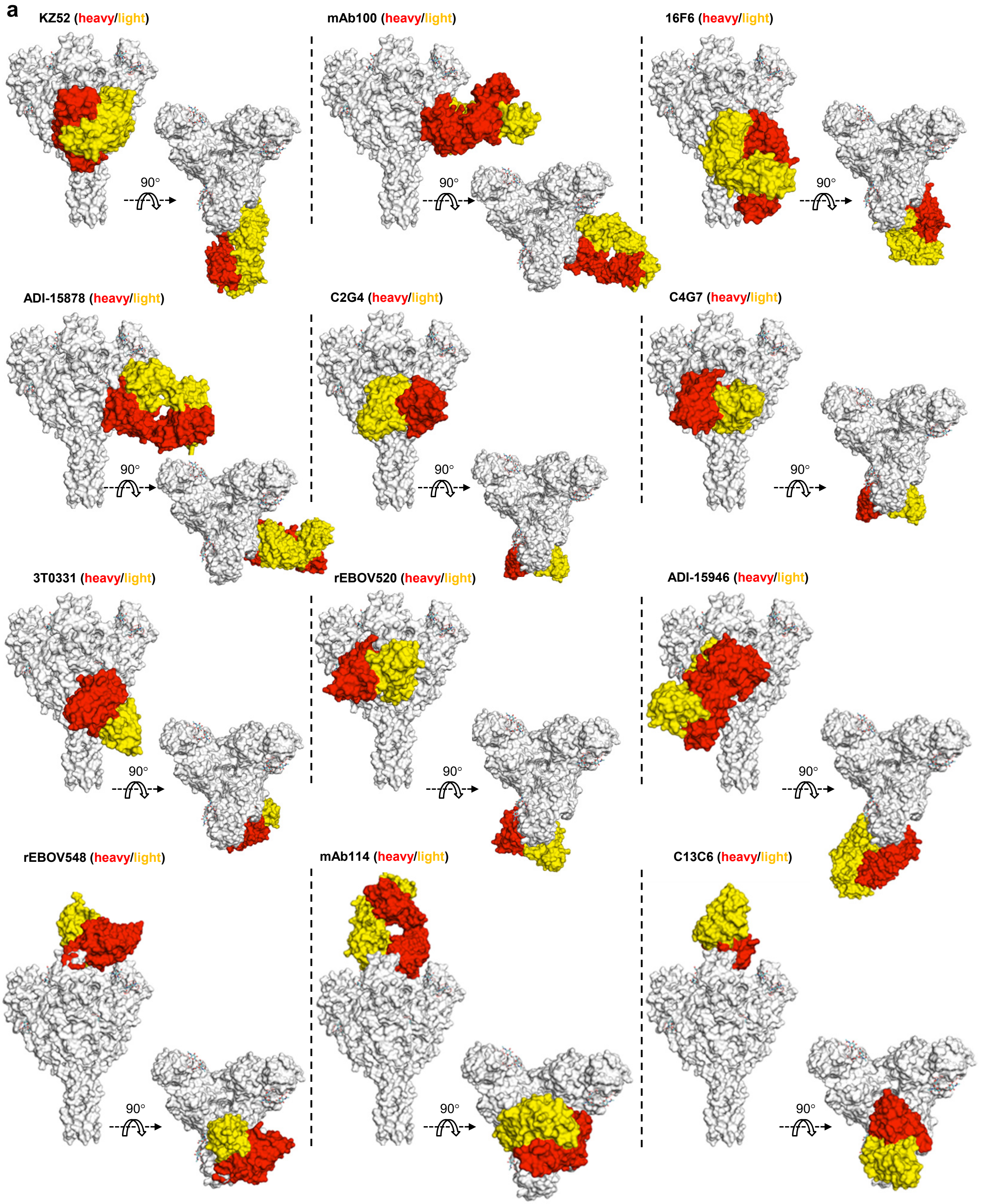
a
KZ52 (heavy/light)
mAb100 (heavy/light)
16F6 (heavy/light)
ADI-15878 (heavy/light)
C2G4 (heavy/light)
C4G7 (heavy/light)
3T0331 (heavy/light)
rEBOV520 (heavy/light)
ADI-15946 (heavy/light)
rEBOV548 (heavy/light)
mAb114 (heavy/light)
C13C6 (heavy/light)
90°

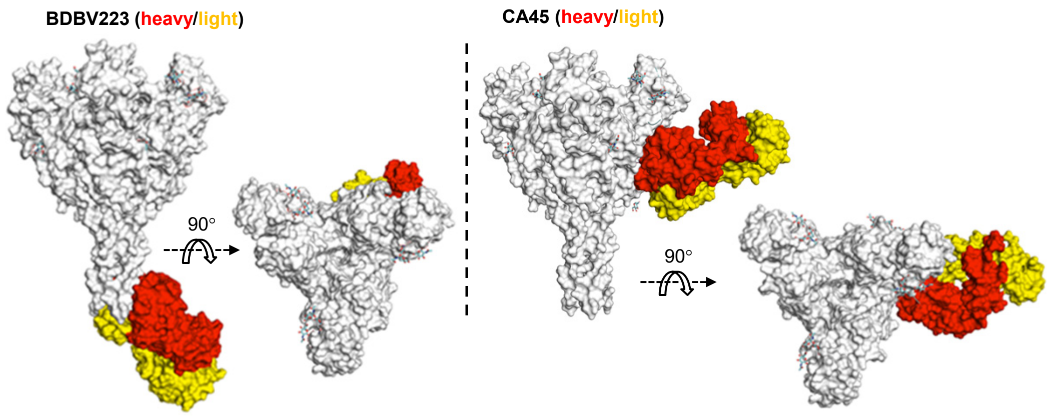

b

```
     32           47           60
5KEN -IPLGVIHNSTLQVSDVDKLVCRDKLSST C4G7
5KEL -IPLGVIHNSTLQVSDVDKLVCRDKLSST C13C6/C2G4
3CSY SIPLGVIHNSVLQVSDVDKLVCRDKLSST KZ52
3S88 SMPLGVVTNSTLEVTEIDQLVCKDHLAST 16F6
6EA5 -IPLGVVHNNTLQVSDIDKLVCRDKLSST ADI-15878
5FHC SIPLGVIHNSTLQVSDVDKLVCRDKLSST mAB114/mAB100
6PCI SIPLGVIHNSTLQVSDVDKLVCRDKLSST rEBOV548/rEBOV520
6MAM SIPLGVIHNSTLQVSDVDKLVCRDKLSST ADI-15946
6QD7 SIPLGVIHNSALQVSDVDKLVCRDKLSST 3T0331
6N7J ----------------------------- BDBV223
6EAY SIPLGVIHNSTLQVSDVDKLVCRDKLSST CA45
            70                             100                 120
5KEN NQLRSVGLNLEGNGVATDVPSATKRWGFRSGVPPKVVNYEAGEWAENCYNLEIKKPDGSE C4G7
5KEL NQLRSVGLNLEGNGVATDVPSATKRWGFRSGVPPKVVNYEAGEWAENCYNLEIKKPDGSE C13C6/C2G4
3CSY NQLRSVGLNLEGNGVATDVPSATKRWGFRSGVPPKVVNYEAGEWAENCYNLEIKKPDGSE KZ52
3S88 DQLKSVGLNLEGSGVSTDIPSATKRWGFRSGVPPKVVSYEAGEWAENCYNLEIKKPDGSE 16F6
6EA5 SQLASVGLNLEGNGVATDVPTATKRWGFRAGVPPKVVNYEAGEWAENCYNLDIKKADGSE ADI-15878
5FHC NQLRSVGLNLEGNGVATDVPSATKRWGFRSGVPPKVVNYEAGEWAENCYNLEIKKPDGSE mAB114/mAB100
6PCI NQLRSVGLNLEGNGVATDVPSVTKRWGFRSGVPPKVVNYEAGEWAENCYNLEIKKPDGSE rEBOV548/rEBOV520
6MAM NQLRSVGLNLEGNGVATDVPSATKRWGFRSGVPPKVVNYEAGEWAENCYNLEIKKPDGSE ADI-15946
6QD7 NQLRSVGLNLEGNGVATDVPSATKRWGFRSGVPPKVVNYEAGEWAENCYNLEIKKPDGSE 3T0331
6N7J ------------------------------------------------------------ BDBV223
6EAY NQLRSVGLNLEGNGVATDVPSATKRWGFRSGVPPKVVNYEAGEWAENCYNLEIKKPDGSE CA45
     136          186                     211                           240
5KEN RYVHKVSGTGPCALPQAKKDFFSSHPLREPVNATEDPSSGYYSTTIRYQATGFGTNETEYLFEVDNLT C4G7
5KEL RYVHKVSGTGPCALPQAKKDFFSSHPLREPVNATEDPSSGYYSTTIRYQATGFGTNETEYLFEVDNLT C13C6/C2G4
3CSY RYVHKVSGTGPCALPQAKKDFFSSHPLREPVNATEDPSSGYYSTTIRYQATGFGTNEVEYLFEVDNLT KZ52
3S88 RYVHKAQGTGPCPLAKPKETFLQSPPIREAVNYTENTSSYYATSYLEYEIENFGAQHSTTLFKIDNNT 16F6
6EA5 RYVHKVSGTGPCPLPETKKDFFEI-------------------------------------------- ADI-15878
5FHC RYVHKVSGTGPCALPQAKKDFFSSHPLREPVNATEDPSSGYYSTTIRYQATGFGTNETEYLFEVDNLT mAB114/mAB100
6PCI RYVHKVSGTGPCALPQAKKDFFSSHPLREPVNATEDPSSGYYSTTIRYQATGFGTNETEYLFEVDNLT rEBOV548/rEBOV520
6MAM RYVHKVSGTGPCALPQAKKDFFSSHPLREPVNATEDPSSGYYSTTIRYQATGFGN------------- ADI-15946
6QD7 RYVHKVSGTGPCALPQAKKDFFSSHPLREPVNATEDPSSGYYSTTIRYQATGFGTNETEYLFEVDNLT 3T0331
6N7J -------------------------------------------------------------------- BDBV223
6EAY RYVHKVSGTGPCALPQAKKDFFSSHPLREPVNATEDPSSGYYSTTIRYQATGFGTNETEYLFEVDNLT CA45
     241                          270                          302
5KEN YVQLESRFTPQFLLQLNETIYTSGKRSNTTGKLIWKVNPEIDTTIGEWAFWETKKNLIRSEELSFA-- C4G7
5KEL YVQLESRFTPQFLLQLNETIYTSGKRSNTTGKLIWKVNPEIDTTIGEWAFWETKKNLIRSEELSFTVV C13C6/C2G4
3CSY YVQLESRFTPQFLLQLNETIYTSGKRSNTTGKLIWKVNPEIDTTIGEWAFWETKKNLIRSEELSFTVV KZ52
3S88 FVRLDRPHTPQFLFQLNDTIHLHQQLSNTTGRLIWTLDANINADIGEWAFWENKKNLLRGEELSFEAL 16F6
6EA5 -------------------------------------------------------------------- ADI-15878
5FHC YVQLESRFTPQFLLQLNETIYTSGKRSNTTGKLIWKVNPEIDTTIGEWAFWETKKNLIRSEELSFSR- mAB114/mAB100
6PCI YVQLESRFTPQFLLQLNETIYASGKRSNTTGKLIWKVNPEIDTTIGEWAFWETKKNLIRSEELSFTN- rEBOV548/rEBOV520
6MAM -------------------------------------------------------------------- ADI-15946
6QD7 YVQLESRFTPQFLLQLNETIYTSGKRSNTTGKLIWKVNPEIDTTIGEWAFWETKKNLIRSEELSFTVV 3T0331
6N7J -------------------------------------------------------------------- BDBV223
6EAY YVQLESRFTPQFLLQLNETIYTSGKRSNTTGKLIWKVNPEIDTTIGEWAFWETKKNLIRSEELSFTVV CA45
     502                         545                         619
5KEN --IVNAQPKCNPNLHYWTTQDEGAAITEGLMHNQDGLICGLRQLANETTQAL---------------- C4G7
5KEL EAIVNAQPKCNPNLHYWTTQDEGAAITEGLMHNQDGLICGLRQLANETTQALTDKIDQIIHDFVDKTL C13C6/C2G4
3CSY EAIVNAQPKCNPNLHYWTTQDEGAAITEGLMHNQDGLICGLRQLANETTQALTDKIDQIIHDFVD--- KZ52
3S88 QTNTKATGKCNPNLHYWTAQEQHNAATEGLMHNQNALVCGLRQLANETTQAL--TSPIVKSFNRN--- 16F6
6EA5 --TLRTQAKCNPNLHYWTTQDEGAAITEGIMHNQNGLICGLRQLANETTQAL---------------- ADI-15878
5FHC EAIVNAQPKCNPNLHYWTTQDEGAAIIEGLMHNQDGLICGLRQLANETTQAL---------------- mAB114/Mab100
6PCI EVIVNAQPKCNPNLHFWTTQDEGAAITEGLMHNQDGLICGLRQLANETTQALTDKIDQIIHDDDDKAG rEBOV548/rEBOV520
6MAM EAIVNAQPKCNPNLHYWTTQDEGAAIIEGLMHNQDGLICGLRQLANETTQAL---------------- ADI-15946
6QD7 EAIVNAQPKCNPNLHYWTTQDEGAAIIEGLMHNQDGLICGLRQLANETTQALTDKIDQIIHDFVDGSG 3T0331
6N7J ----------------------------------------------------TDKIDQIIHDFIDKPL BDBV223
6EAY EAIVNAQPKCNPNLHYWTTQDEGAAITEGLMHNQDGLICGLRQLANETTQALTDKIDQIIHDFVDKTL CA45
```

GP1 (brace spanning residues 32–302 blocks)

GP2 (brace spanning residues 502–619 block)

Fig. S5. Structural analysis of known GP/antibody complexes. (**a**) Molecular surface representation of the GPΔmuc-WL^{2}P^2 trimer bound to antibodies by docking the crystal structure of GPΔmuc-WL^{2}P^2-foldon (grey) into previously determined GP/antibody complex structures (Left: side view; Right: top view). Antibody heavy and light chains are shown in red and yellow, respectively. (**b**) Sequence analysis of antibody-contacting residues (≤ 4.0 Å) in GP1 and GP2. In the ClustalW alignment, antibody-contacting residues are colored in green except for 5KEL, 5FHC, and 6PCI, for which residues interacting with the first and second antibodies are colored in red and cyan, respectively, to define their respective epitopes.

a

Dimeric locking domains (LD) for stabilizing the NP-forming interface

>**LD1** 1NI8_A (**40aa** after C-terminal deletion)
SEALKILNNIRTLRAQARECTLETLEEMLEKLEVVVNERR~~**EEESAA**~~

>**LD2** 4AYA_B (**44aa** after N-/C-terminal deletion)
MNDCYSKLKELVPSIPQNKKVSKMEILQHVIDYILDLQIALDSH

>**LD3** 1OVX_A (**50aa** after N-terminal deletion)
~~**GHHHHHHTDKRKDGSGK**~~LLYCSFCGKSQHEVRKLIAGPSVYICDECVDLCNDIIREEIKEVAPHRER

>**LD4** 2MG4_A (**58aa** after N-terminal deletion)
~~**MEKRPRTE**~~FSEEQKKALDLAFYFDRRLTPEWRRYLSQRLGLNEEQIERWFRRKEQQIGWSHPQFEK

>**LD5** 2JV7_A (**78aa** without deletion)
DQPSVGDAFDKYNEAVRVFTQLSSAANCDWAACLSSLSASSAACIAAVGELGLDVPLDLACAATATSSATEACKGCLW

>**LD6** 1JR5_A (**88aa** after N-terminal deletion)
~~**MN**~~KNIDTVREIITVASILIKFSREDIVENRANFIAFLNEIGVTHEGRKLNQNSFRKIVSELTQEDKKTLIDEFNEGFEGVYRYLEMYTNK

>**LD7** 1PZQ_A (**46aa** after N-/C-terminal deletion)
~~**GSAA**~~SPAVDIGDRLDELEKALEALSAEDGHDDVGQRLESLLRRWNSRRAD~~**APSTSAISED**~~

>LD8 1R2A_A (**39aa** after N-terminal deletion)
~~**HMGHIQI**~~PPGLTELLQGYTVEVLRQQPPDLVDFAVEYFTRLREARR

>LD9 2JRX_A (**65aa** after N-/C-terminal deletion and glycan deletion)
~~**MPQISR**~~YSDEQVEQLLAELLNVLEKHKAPTDLSLMVLGNMVTNLINT**A**IAPAQRQAIANSFARALQSSINE~~**DKAHLEHHHHHH**~~

b

Antigenic evaluation of NPs presenting a stabilized GPΔmuc trimer

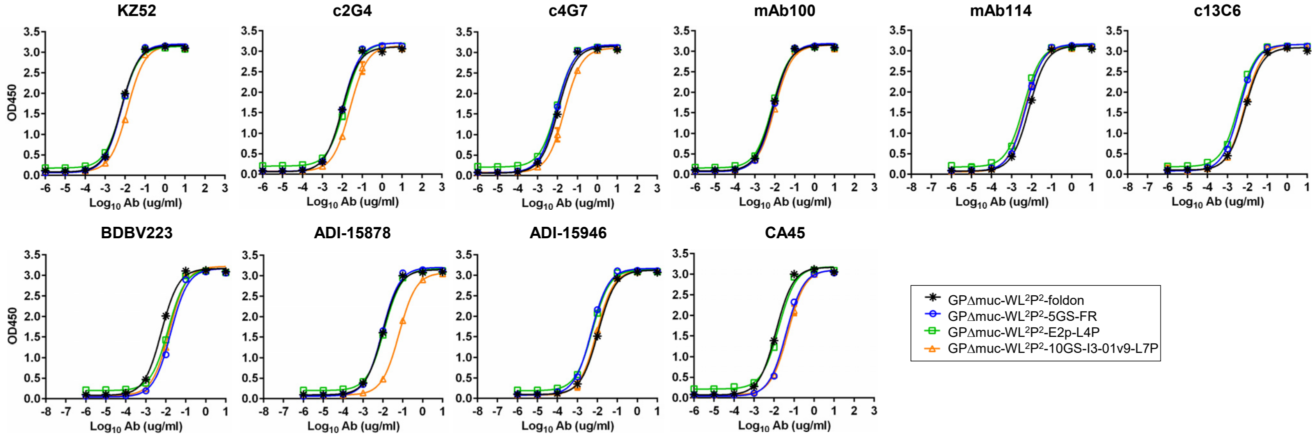

c

EC_{50} values (μg/ml) of EBOV GPΔmuc trimer and nanoparticles binding to 10 antibodies [a].

	KZ52	c2G4	c4G7	mAb100	mAb114	c13C6	BDBV223	ADI-15878	ADI-15946	CA45
GPΔmuc-WL^{2}P^2-foldon	0.006	0.010	0.011	0.008	0.007	0.008	0.006	0.010	0.011	0.012
GPΔmuc-WL^{2}P^2-5GS-FR	0.007	0.010	0.009	0.009	0.005	0.004	0.018	0.009	0.005	0.040
GPΔmuc-WL^{2}P^2-E2p-L4P	0.007	0.013	0.009	0.008	0.004	0.004	0.013	0.012	0.006	0.017
GPΔmuc-WL^{2}P^2-10GS-I3-01v9-L7P	0.013	0.024	0.023	0.010	0.005	0.007	0.015	0.065	0.009	0.049

[a] EC_{50} values were calculated from the besting fitting in GraphPad Prism 8.4.3.

d GPΔmuc-WL^{2}P^2-foldon

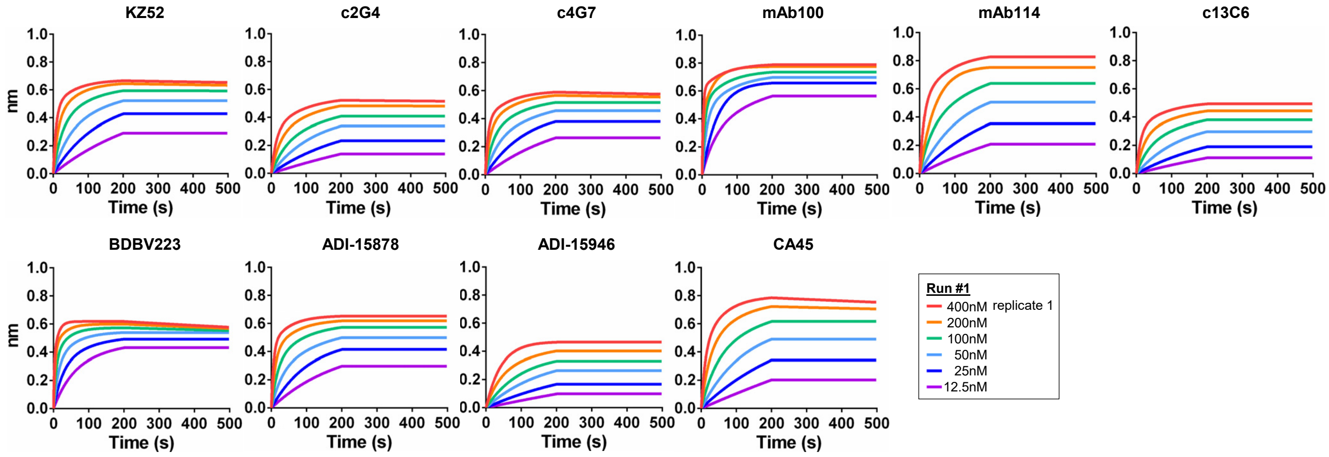

GPmmuc-WL^{2}P^2-5GS-FR

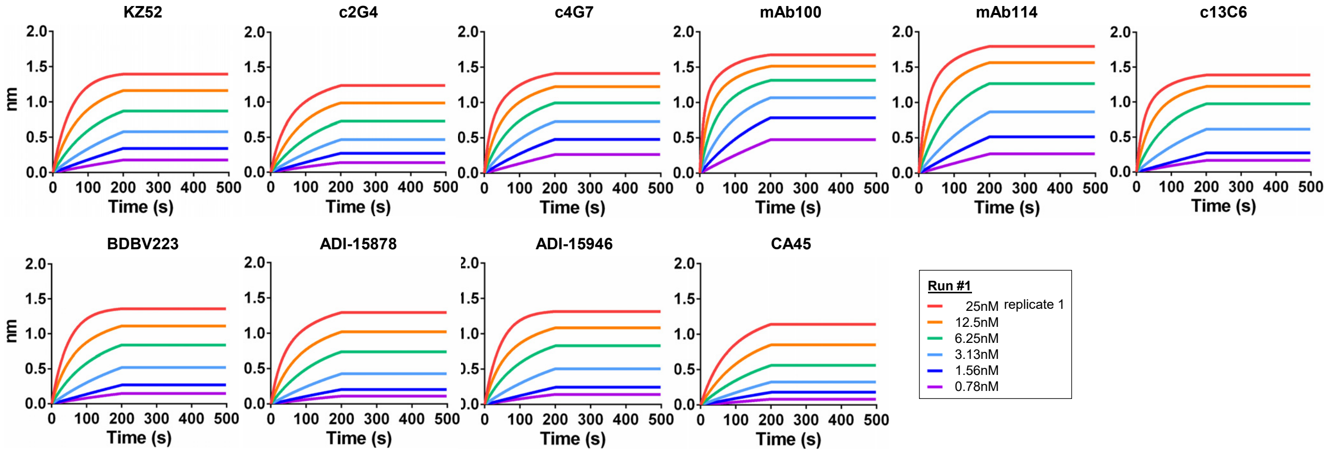

GPΔmuc-WL^{2}P^2-E2p-L4P

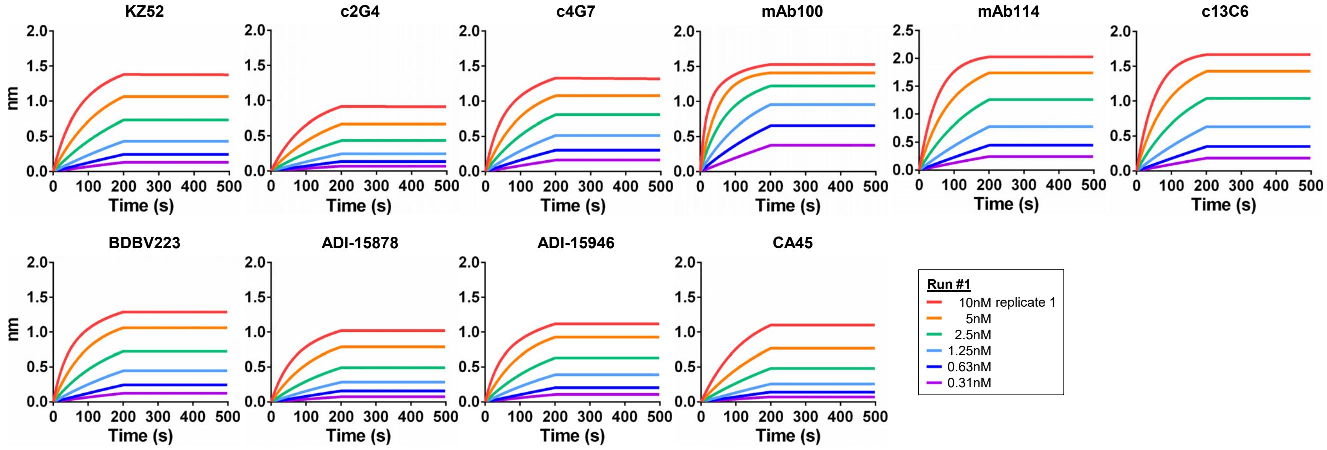

Fig. S6

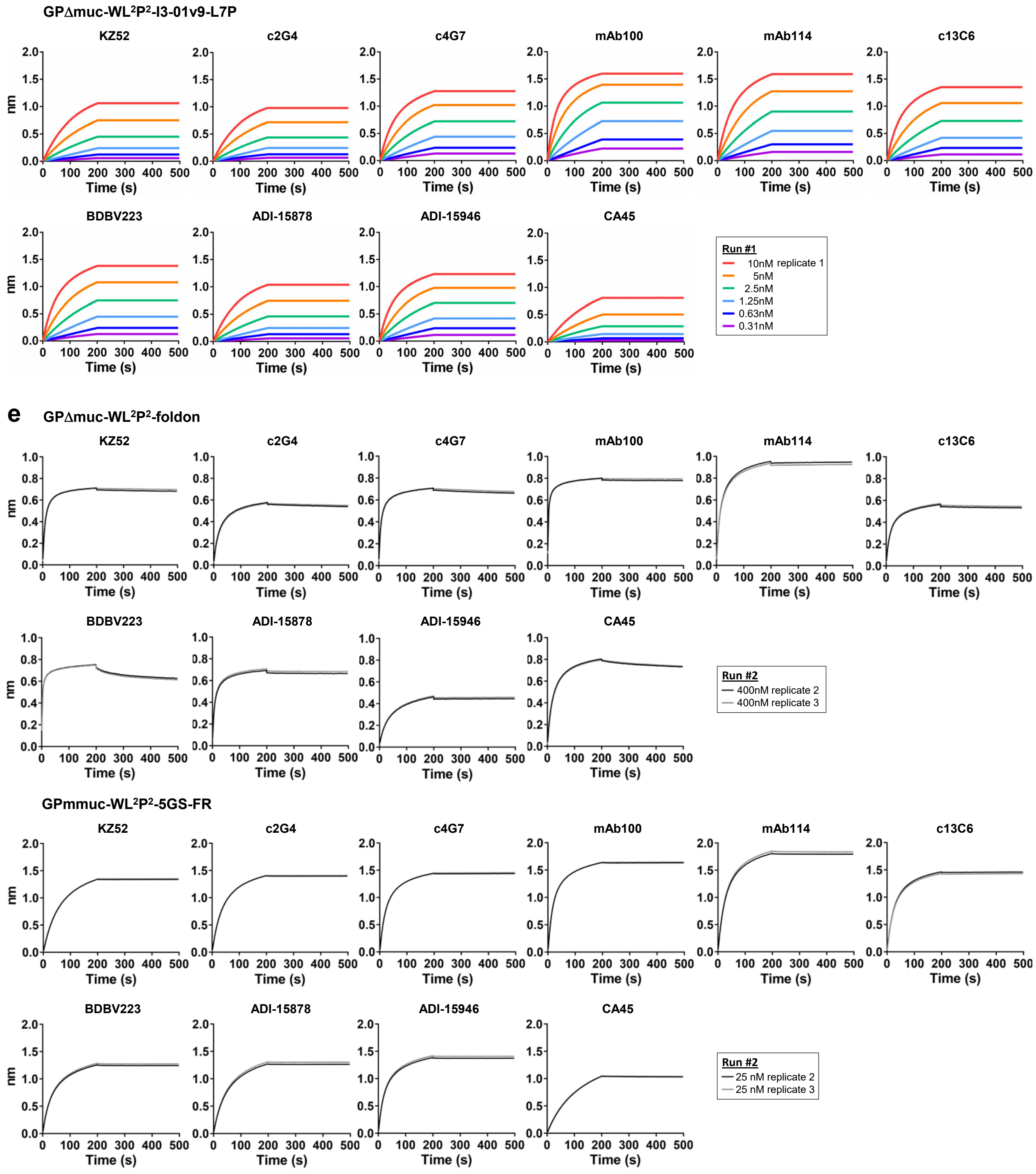
GPΔmuc-WL2P2-I3-01v9-L7P
KZ52
c2G4
c4G7
mAb100
mAb114
c13C6
BDBV223
ADI-15878
ADI-15946
CA45
nm
Time (s)
Run #1
10nM replicate 1
5nM
2.5nM
1.25nM
0.63nM
0.31nM
e
GPΔmuc-WL2P2-foldon
Run #2
400nM replicate 2
400nM replicate 3
GPmmuc-WL2P2-5GS-FR
Run #2
25 nM replicate 2
25 nM replicate 3

Fig. S6

GPΔmuc-WL²P²-E2p-L4P

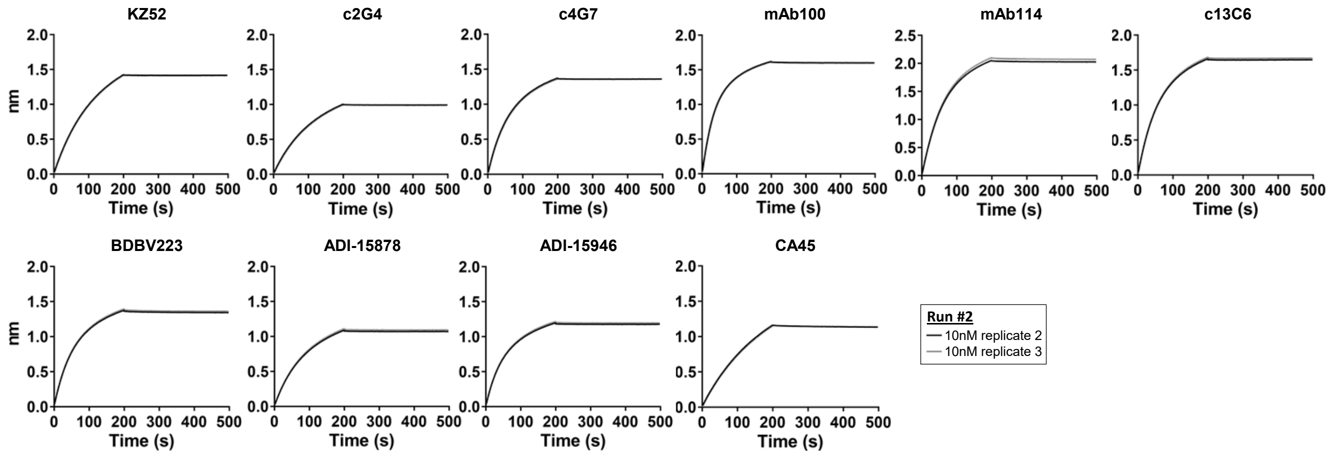

GPΔmuc-WL²P²-I3-01v9-L7P

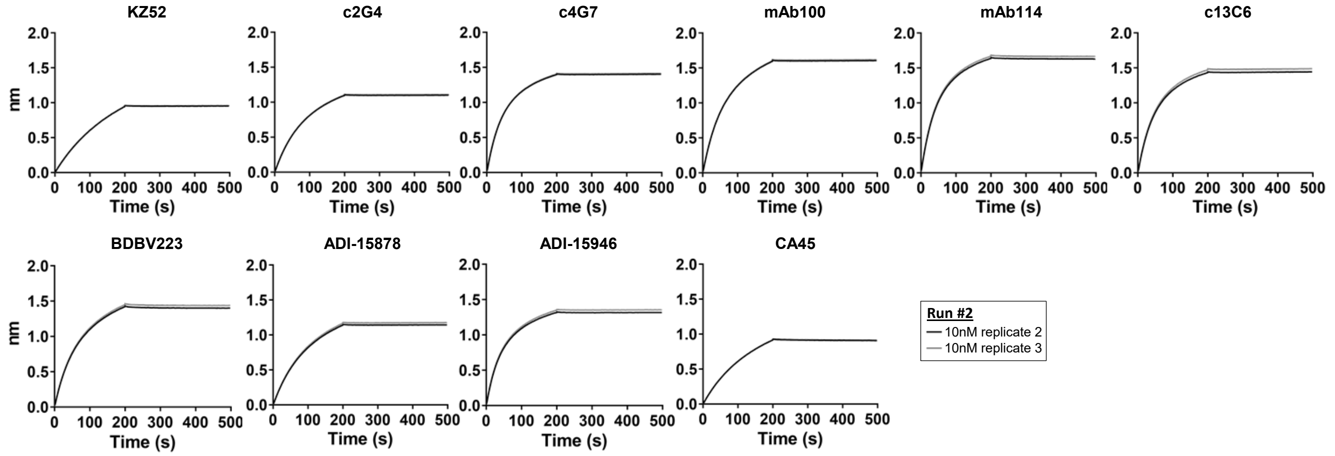

f Standard deviation of antibody binding signals at the highest antigen concentration (3 replicates)

EBOV GP constructs:	KZ52	c2G4	c4G7	mAb100	mAb114	c13C6	BDBV223	ADI-15878	ADI-15946	CA45
GPΔmuc-WL²P²-foldon	0.02	0.03	0.07	0.01	0.06	0.04	0.07	0.02	0.01	0.00
GPΔmuc-WL²P²-5GS-FR	0.04	0.09	0.02	0.03	0.03	0.03	0.06	0.02	0.04	0.06
GPΔmuc-WL²P²-E2p-L4P	0.02	0.04	0.02	0.05	0.03	0.02	0.05	0.03	0.04	0.02
GPΔmuc-WL²P²-10GS-I3-01v9-L7P	0.08	0.06	0.06	0.01	0.03	0.05	0.03	0.06	0.05	0.06

g mAb100-purified GP_{ECTO}-10GS-FR

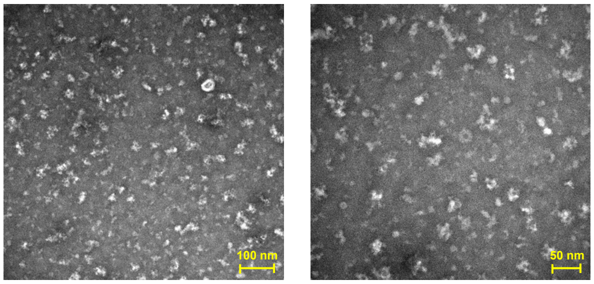

Fig. S6. Design and characterization of multilayered EBOV GPΔmuc-presenting NPs. (**a**) Locking domains (LD) selected from 815 homodimers in the PDB for stabilizing the NP-forming interface. PDB IDs, number of residues after N- and C-terminal deletion, and amino acid sequences are provided. (**b**)-(**c**) Antigenic evaluation by ELISA. Binding of FR and reengineered 60-meric E2p (E2p-LD4-PADRE, or E2p-L4P) and I3-01v9 (I3-01v9-LD7-PADRE, or I3-01v9-L7P) NPs that present a stabilized GPΔmuc-WL^{2}P^2 trimer to 10 antibodies with ELISA curves shown in (**b**) and EC_{50} values (μg/ml) summarized in (**c**). All GP-NPs were expressed transiently in ExpiCHO cells, purified by a mAb100 column, and further purified by SEC on a Superose 6 10/300 GL column. (**d**) Antigenic evaluation of GPΔmuc-WL^{2}P^2-foldon and three NPs (FR, E2p-L4P, and I3-01v9-L7P) that present GPΔmuc-WL^{2}P^2 using BLI and 10 representative antibodies. This experiment is designated "Run #1". Sensorgrams were obtained from an Octet RED96 instrument using six concentrations (400-12.5 nM, 25-0.78 nM, and 10-0.31 nM by twofold dilution for GPΔmuc, FR, and E2p-L4p/I3-01v9-L7P, respectively) and AHQ biosensors (see Methods). I3-01v9 is a variant of I3-01 with a redesigned NP-forming interface based on the PDB structure (1VLW). I3-01v9 was reported in our previous study (ref. 55) with a construct name "1VLW-v9". (**e**) Antigenic evaluation of GPΔmuc-WL^{2}P^2-foldon and three NPs (FR, E2p-L4P, and I3-01v9-L7P) that present GPΔmuc-WL^{2}P^2 using BLI and 10 representative antibodies at the highest antigen concentration in (**d**) with two replicates. This experiment is designated "Run #2". (**f**) Standard deviation of antibody binding signals at the highest concentration using 3 replicates from Run #1 and Run #2. Color coding is based on the range of binding signals shown in Fig. 4j. (**g**) Representative micrographs from the negative-stain EM of GP_{ECTO}-10GS-FR following ExpiCHO expression and mAb114 purification. The use of mAb114 instead of mAb100 for IAC purification was based on the consideration that the MLD may occlude antibody access to IFL on the FR NP surface. Source data are provided as a Source Data file.

Fig. S7

a

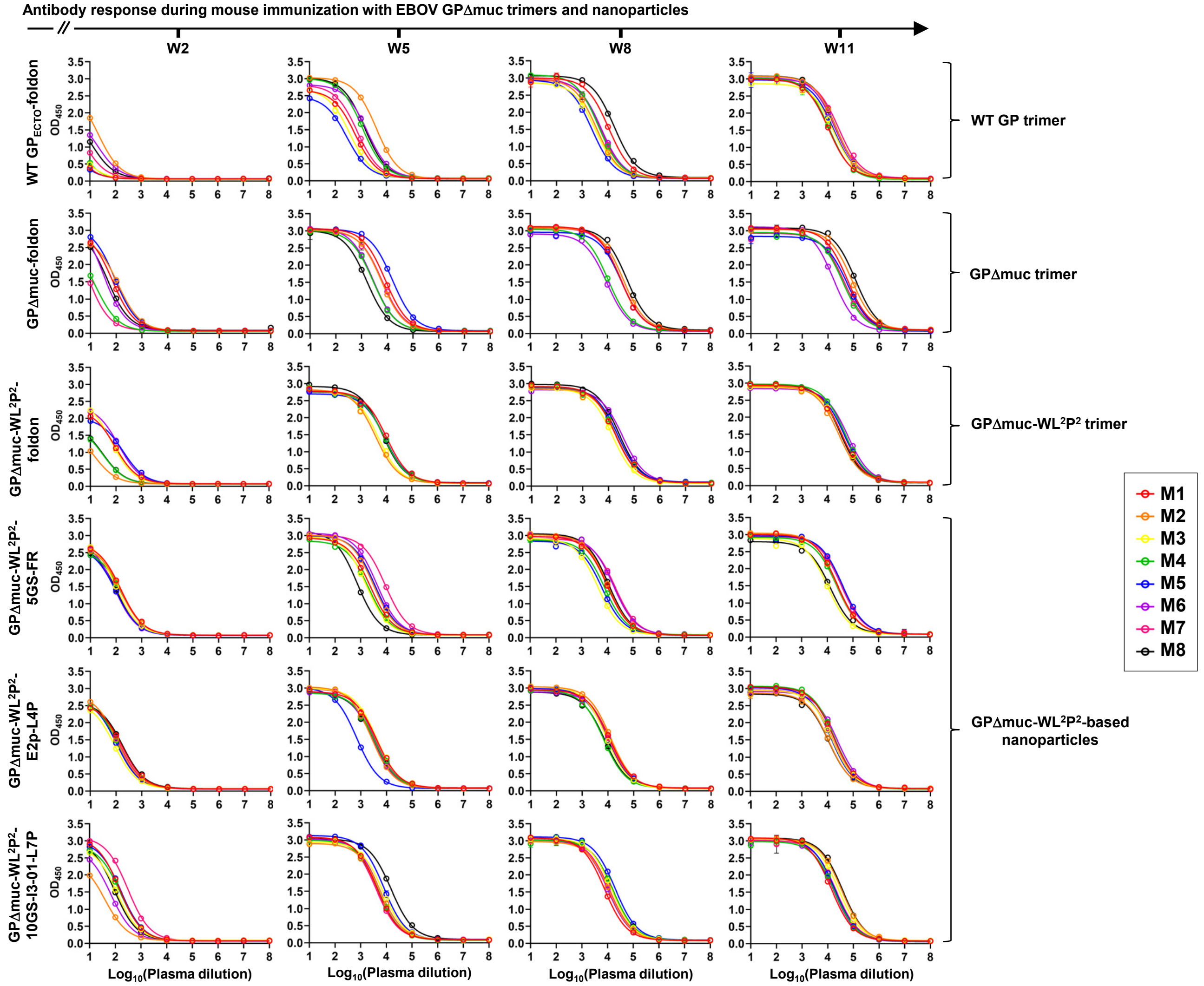

b

EC_{50} titers (fold of dilution) of mouse plasma binding to EBOV GP trimers[a]

	W2			W5			W8			W11		
	WT GP_{ECTO}-foldon	GPΔmuc-foldon	GPΔmuc-WL^2P^2-foldon	WT GP_{ECTO}-foldon	GPΔmuc-foldon	GPΔmuc-WL^2P^2-foldon	WT GP_{ECTO}-foldon	GPΔmuc-foldon	GPΔmuc-WL^2P^2-foldon	WT GP_{ECTO}-foldon	GPΔmuc-foldon	GPΔmuc-WL^2P^2-foldon
M1	<1	71	93	607	8377	10280	10740	32034	18532	10467	56041	36300
M2	21	119	14	4050	5879	3894	3663	40677	20082	20353	83513	28303
M3[b]	5	–	67	376	–	4851	3323	–	14595	16418	–	37914
M4	1	16	31	1183	2755	8470	4338	10690	19570	11262	37135	49211
M5	3	89	187	289	15768	8994	2382	35240	22712	16546	56785	46695
M6	20	31	127	1804	2752	8816	5651	9447	35455	18888	16351	64055
M7[b]	5	7	–	740	6293	–	5066	33547	–	26293	42415	–
M8	12	46	31	1449	1591	7248	17622	56265	25518	20626	117745	33217

[a] The EC_{50} values were calculated from the besting fitting in GraphPad Prism 8.4.3.

[b] "-" indicates animal death during the immunization study.

Fig. S7

c

EC_{50} titers (fold of dilution) of mouse plasma binding to EBOV GPΔmuc-WL²P²-presenting nanoparticles [a]

	W2			W5			W8			W11		
	GPΔmuc-WL²P²-5GS-FR	GPΔmuc-WL²P²-E2p-L4P	GPΔmuc-WL²P²-10GS-I3-01v9-L7P	GPΔmuc-WL²P²-5GS-FR	GPΔmuc-WL²P²-E2p-L4P	GPΔmuc-WL²P²-10GS-I3-01v9-L7P	GPΔmuc-WL²P²-5GS-FR	GPΔmuc-WL²P²-E2p-L4P	GPΔmuc-WL²P²-10GS-I3-01v9-L7P	GPΔmuc-WL²P²-5GS-FR	GPΔmuc-WL²P²-E2p-L4P	GPΔmuc-WL²P²-10GS-I3-01v9-L7P
M1	153	172	148	2367	4154	4389	9932	11715	7637	24325	17631	13984
M2	146	112	41	3115	2559	5927	10324	12671	11689	22640	11150	34464
M3	102	86	115	1691	4077	7196	4239	8701	14184	10166	15309	26621
M4	159	121	159	2053	3426	4860	7750	6774	16144	23135	18605	18439
M5	99	106	165	3598	692.1	8006	6227	9166	19698	38846	13793	19315
M6 [b]	84	105	67	4238	2973	5440	17428	12140	10331	–	24189	17649
M7	101	142	305	9029	3443	3872	20184	10342	11975	34598	15778	21201
M8	104	178	93	725.6	3022	15325	11421	7550	16234	13170	11012	37636

[a] The EC_{50} values were calculated from the besting fitting in GraphPad Prism 8.4.3.
[b] "-" indicates animal death during the immunization study.

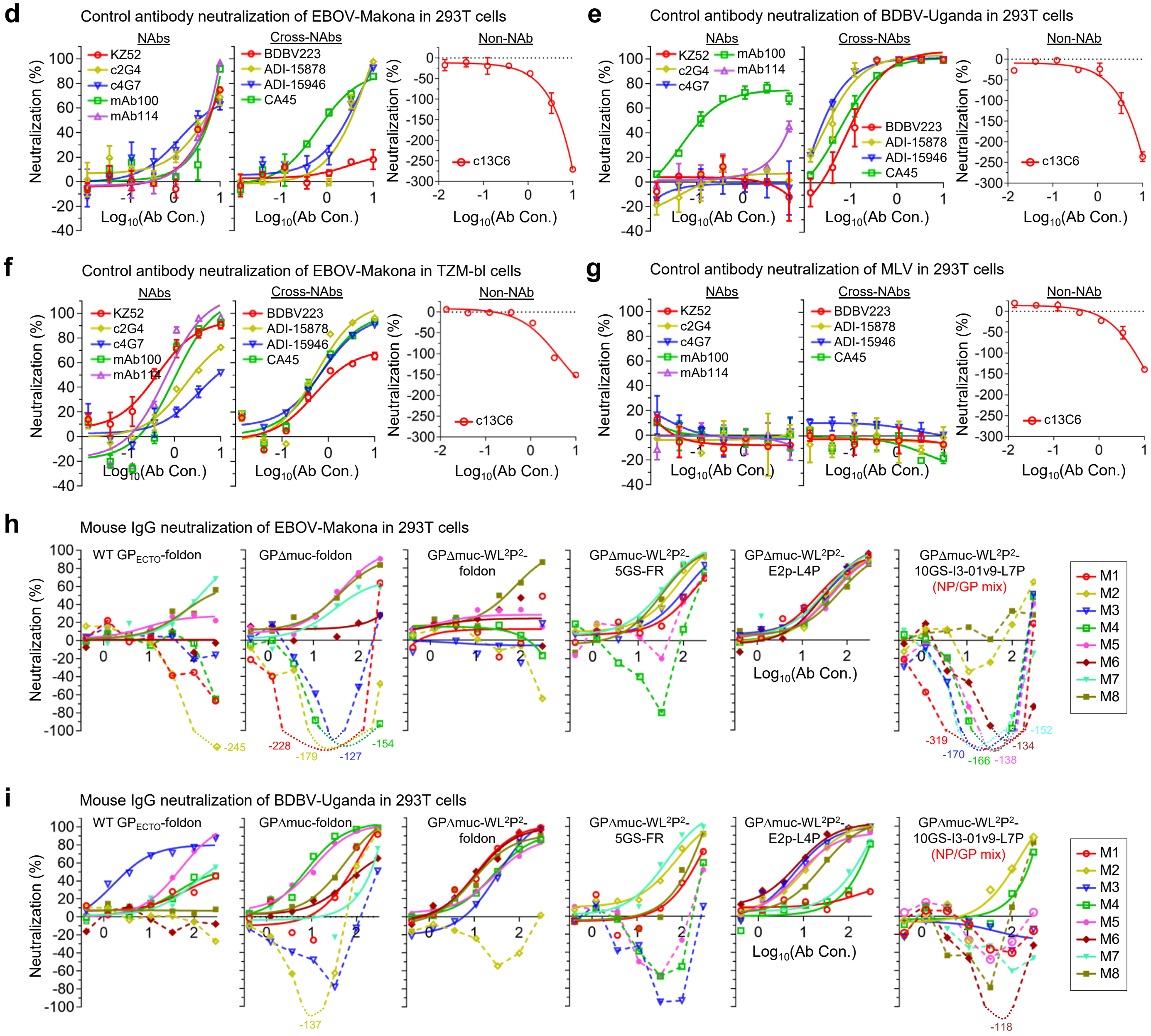

j

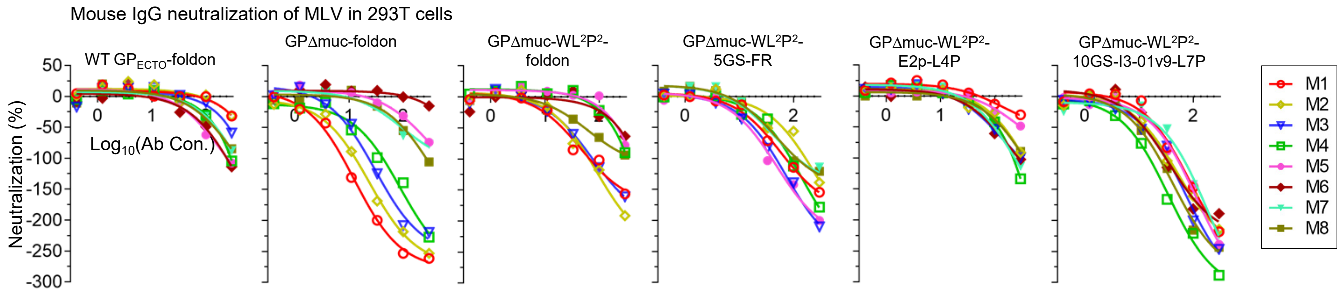

Fig. S7. Immunogenicity of EBOV GP/GPΔmuc trimers and GPΔmuc-presenting NPs in BALB/c mice. (**a**) ELISA binding curves of mouse plasma from three trimer groups, in which mice were immunized with WT GP_{ECTO}-foldon, GPΔmuc-foldon, and GPΔmuc-WL^2P^2-fold trimers, and three NP groups, in which mice were immunized with FR, E2p-L4P, and I3-01v9-L7P NPs presenting GPΔmuc-WL^2P^2, to GPΔmuc-WL^2P^2 at w2, w5, w8, and w11. (**b**) EC_{50} titers measured for the three trimer groups. (**c**) EC_{50} titers measured for the three NP groups. The EC_{50} titer was measured in the unit of fold of dilution. Of note, plasma binding at w2 did not reach the plateau (or saturation) to allow for accurate determination of EC_{50} titers. Nonetheless, the EC_{50} values calculated in Prism were used as a quantitative measure of binding antibody titers to facilitate the comparison of different vaccine groups at w2. (**d**) and (**e**) Ebolavirus-pp (EBOV-Makona and BDBV-Uganda) neutralization by 10 representative antibodies in HEK293T cells. (**f**) Ebolavirus-pp (EBOV-Makona) neutralization by 10 representative antibodies in TZM-bl cells. (**g**) MLV-pp neutralization by 10 representative antibodies in HEK293T cells as a negative control. In (**d**)-(**g**), the antibody neutralization was performed in duplicates, with mean value and standard deviation (SD) shown as symbol and solid line, respectively. (**h**) and (**i**) Ebolavirus-pp (EBOV-Makona and BDBV-Uganda) neutralization by purified mouse IgG from six vaccine groups in HEK293T cells, with (**j**) MLV-pp included as a negative control. The IgG concentration was started at 300μg/ml followed by a series of threefold dilutions. Due to the limited availability of mouse samples, ebolavirus-pp assays were performed only once without duplicate. For mice showing enhanced ebolavirus-pp infection (e.g. %neuralization<-30.0%), data points are shown as dashed lines instead of fitted curves. Data points below -100% are excluded with the lowest %neutralization values labeled on the plot and color-coded according to the mouse index. Mouse IgGs were purified from plasma collected at the last time point, w11. Source data are provided as a Source Data file.

Fig. S8

a

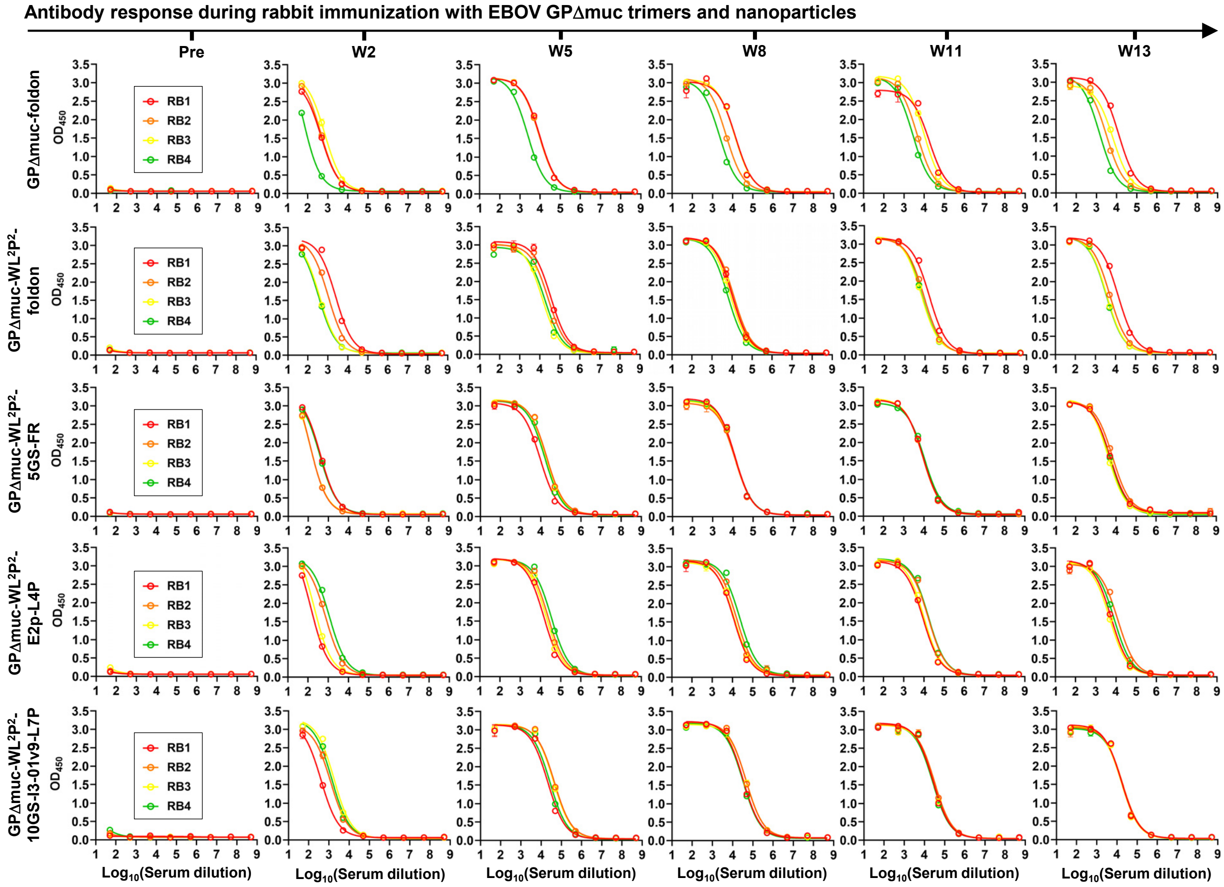

b

EC$_{50}$ titers (fold of dilution) of rabbit sera binding to EBOV GP trimers[a]

	W2		W5		W8		W11		W13	
	GPΔmuc-foldon	GPΔmuc-WL^{2}P^2-foldon	GPΔmuc-foldon	GPΔmuc-WL^{2}P^2-foldon	GPΔmuc-foldon	GPΔmuc-WL^{2}P^2-foldon	GPΔMuc-foldon	GPΔmuc-WL^{2}P^2-foldon	GPΔmuc-foldon	GPΔmuc-WL^{2}P^2-foldon
RB1	464	2428	9404	35115	13868	10110	18135	16116	12777	13338
RB2	456	1044	8904	26144	4647	12073	4446	8694	3143	5177
RB3	701	378	8943	13273	12652	8363	9807	6517	6681	3586
RB4	57	336	2392	18008	2220	6092	2663	6901	1443	3424

[a] The EC$_{50}$ values were calculated from the besting fitting in GraphPad Prism 8.4.3.

EC$_{50}$ titers (fold of dilution) of rabbit sera binding to EBOV WT GPΔmuc-presenting nanoparticles [a]

	W2			W5			W8			W11		
	GPΔmuc-5GS-FR	GPΔmuc-E2p-L4P	GPΔmuc-10GS-I3-01v9-L7P	GPΔmuc-5GS-FR	GPΔmuc-E2p-L4P	GPΔmuc-10GS-I3-01v9-L7P	GPΔmuc-5GS-FR	GPΔmuc-E2p-L4P	GPΔmuc-10GS-I3-01v9-L7P	GPΔmuc-5GS-FR	GPΔmuc-E2p-L4P	GPΔmuc-10GS-I3-01v9-L7P
RB1	393	133	404	9406	14736	21261	12961	11405	32895	9041	8972	26363
RB2	117	735	1207	20093	23950	44607	12927	15609	45311	8893	16560	31073
RB3	120	201	1774	18894	19727	41440	12770	12457	47664	8644	9954	28526
RB4	368	1126	1382	16116	34695	27116	13140	22771	32291	10558	16803	25205

	W13		
	GPΔmuc-5GS-FR	GPΔmuc-E2p-L4P	GPΔmuc-10GS-I3-01v9-L7P
RB1	5272	5795	16907
RB2	6817	12387	18398
RB3	4146	4889	16645
RB4	5047	8066	18753

The EC$_{50}$ values were calculated from the besting fitting in GraphPad Prism 8.4.3.

Fig. S8

c **Ebolavirus-pp neutralization by purified rabbit IgG at the 5th time point (w11)**

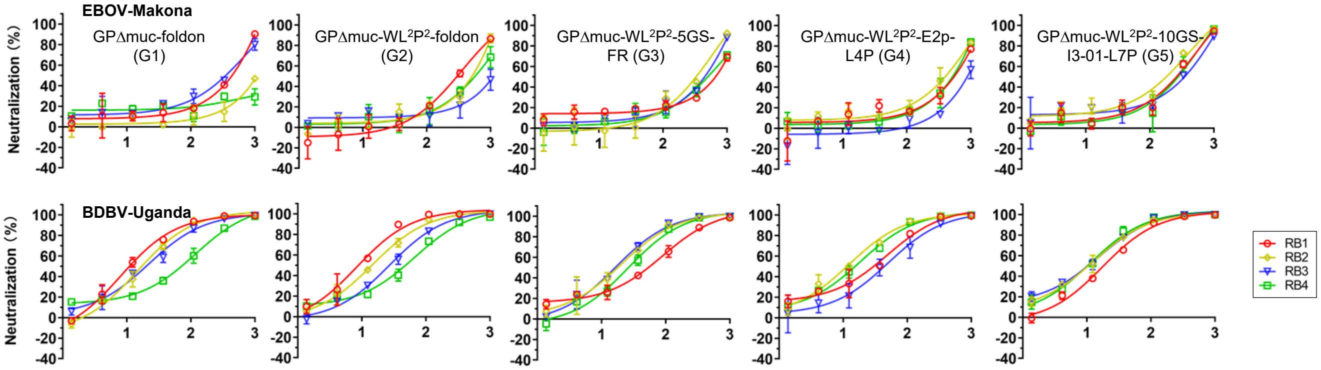

MLV-pp neutralization by purified rabbit IgG at the 5th time point (w11)

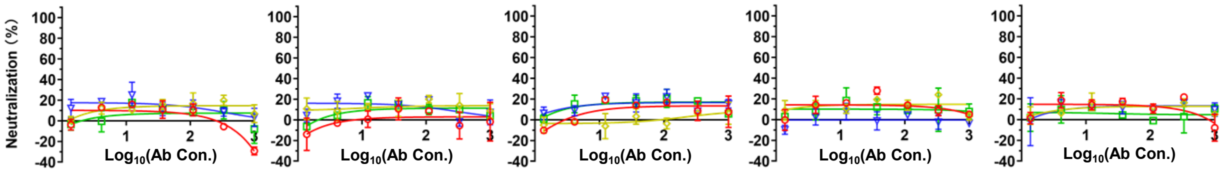

d **Ebolavirus-pp neutralization by purified rabbit IgG at the 1st time point (Pre)**

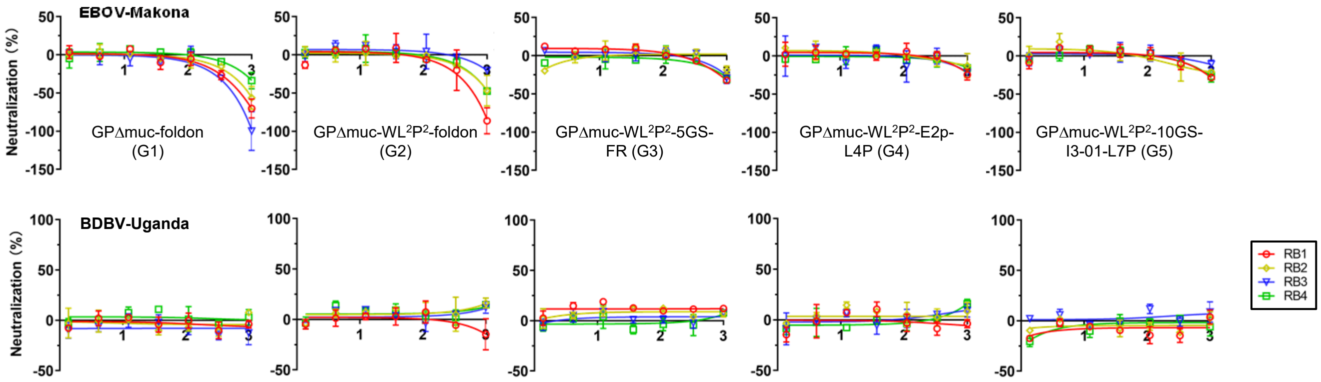

MLV-pp neutralization by purified rabbit IgG at the 1st time point (Pre)

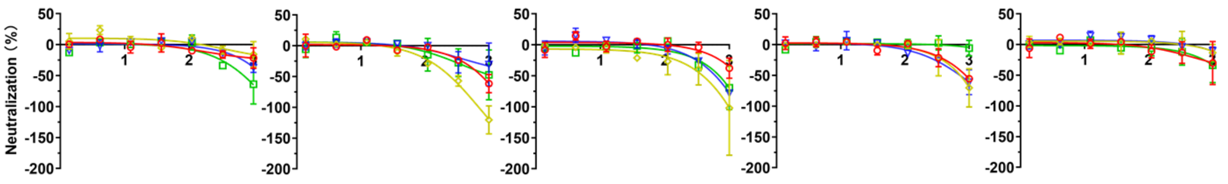

Fig. S8

e **Ebolavirus-pp neutralization by purified rabbit IgG at the 2nd time point (w2)**

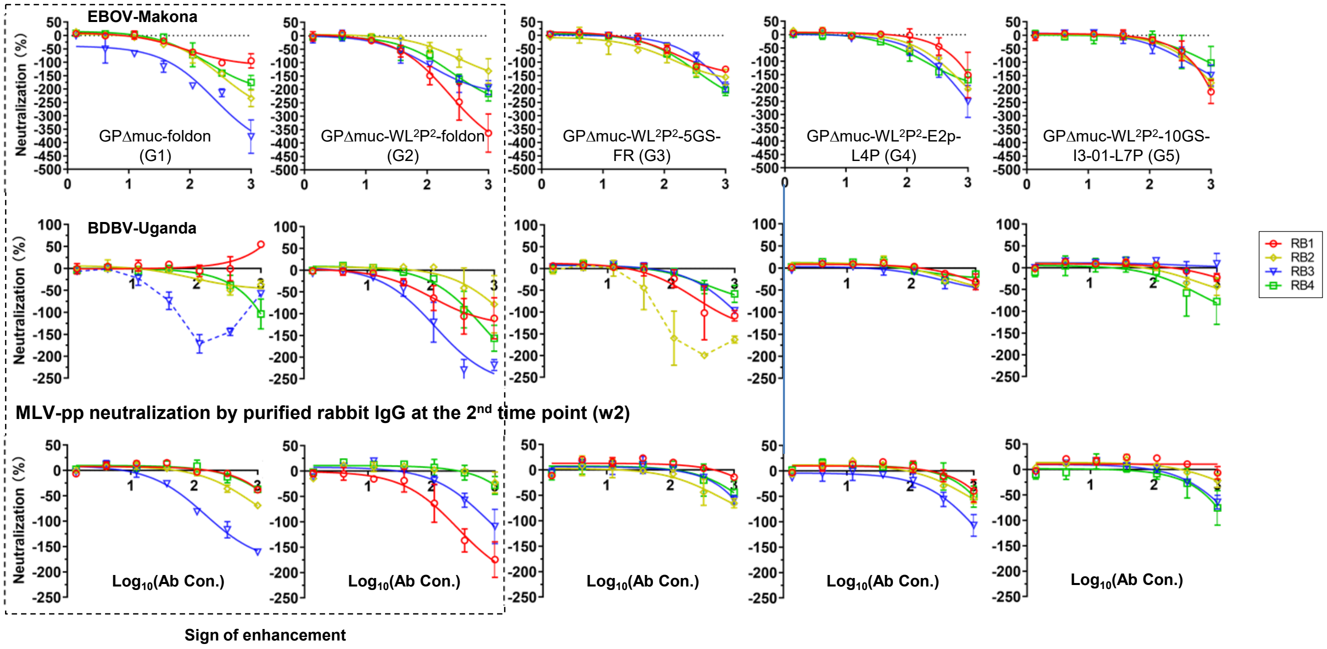

f **Ebolavirus-pp neutralization by purified rabbit IgG at the 3rd time point (w5)**

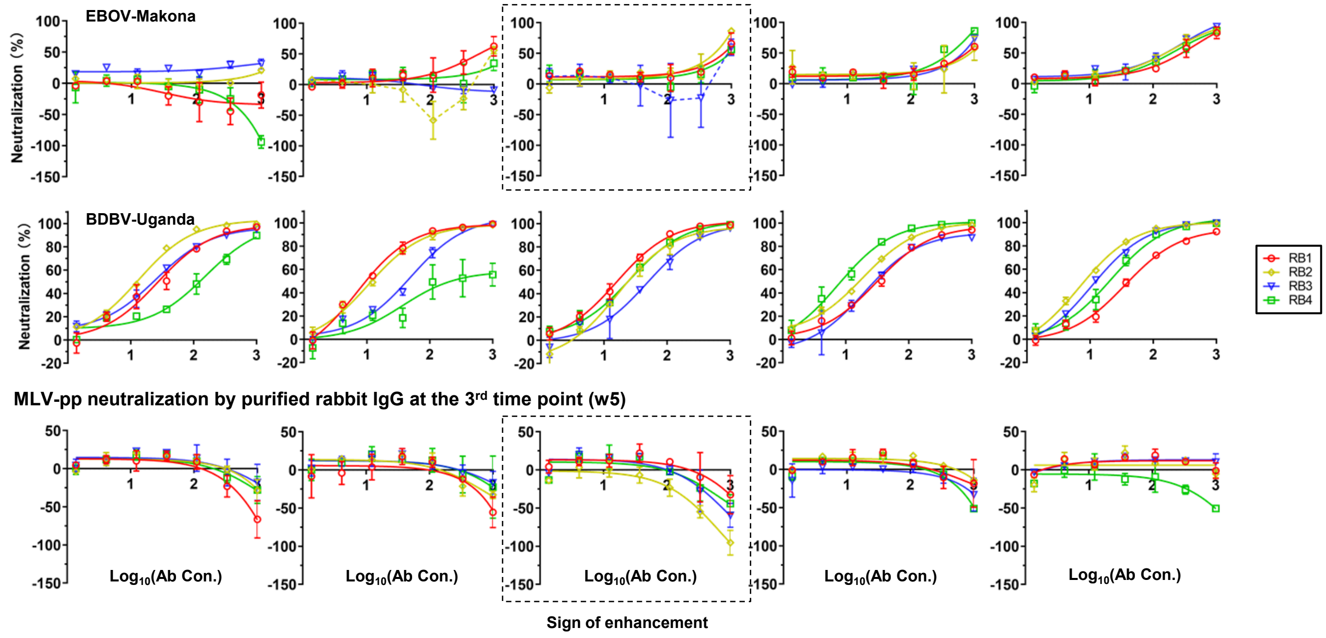

Fig. S8

g **Ebolavirus-pp neutralization by purified rabbit IgG at the 4th time point (w8)**

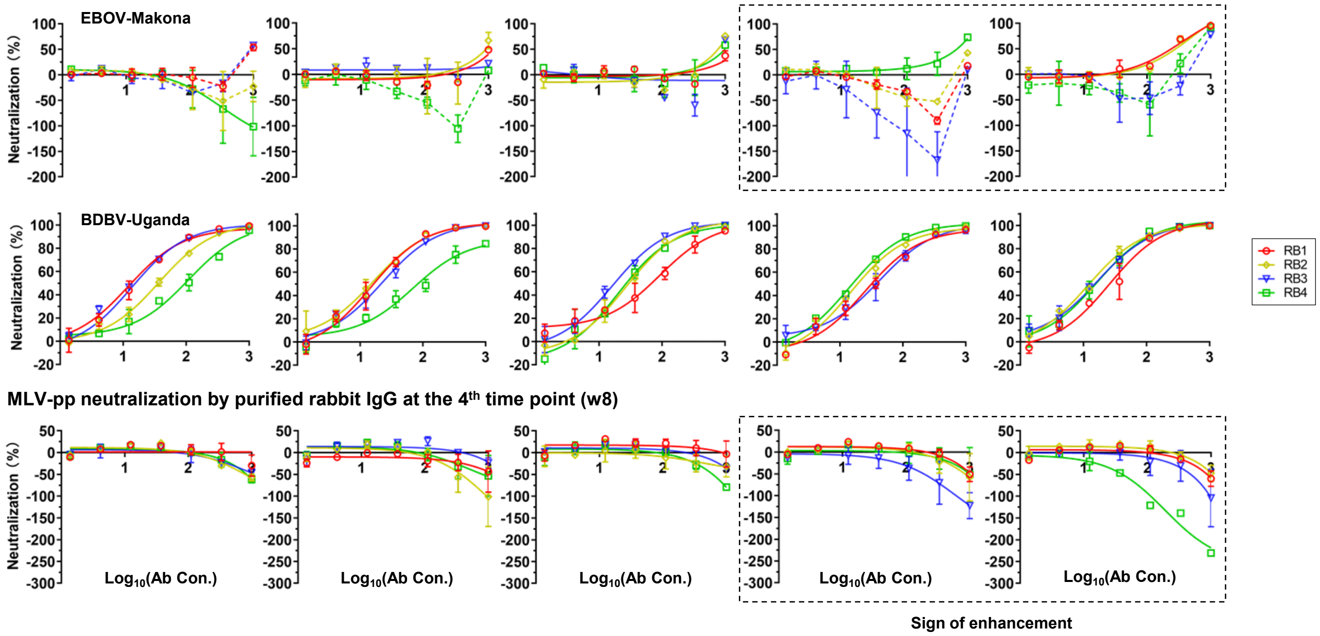

Fig. S8. Immunogenicity of EBOV GP/GPΔmuc trimers and GPΔmuc-presenting NPs in rabbits. (**a**) ELISA binding curves of rabbit sera from two trimer groups, in which rabbits were immunized with GPΔmuc-foldon and GPΔmuc-WL^{2}P^2-fold trimers, and three NP groups, in which rabbits were immunized with FR, E2p-L4P, and I3-01v9-L7P NPs presenting GPΔmuc-WL^{2}P^2, to GPΔmuc-WL^{2}P^2 at w0, w2, w5, w8, w11, and w13. (**b**) EC_{50} titers measured for the two trimer groups and three NP groups. The EC_{50} titer was measured in the unit of fold of dilution. Of note, serum binding at w2 did not reach the plateau (or saturation), but the OD_{450} values were approximately at the same level as those obtained at w5 (fully plateaued). Therefore, the EC_{50} values calculated in Prism were considered sufficiently accurate to facilitate the comparison of different vaccine groups at w2. (**c**) Ebolavirus-pp (EBOV-Makona and BDBV-Uganda) and MLV-pp neutralization by purified rabbit IgGs from w11 in HEK293T cells. No sign of enhanced pseudovirus infection is observed. Ebolavirus-pp (EBOV-Makona and BDBV-Uganda) and MLV-pp neutralization by purified rabbit IgG from Pre, w2, w5, and w8 in HEK293T cells is shown in (**d**), (**e**), (**f**) and (**g**). In (**c**)-(**g**), the rabbit IgG neutralization was performed in duplicates, with mean value and standard deviation (SD) shown as symbol and solid line, respectively. Data points that do not fit a sigmodal dose response model are shown as dashed lines. Signs of enhanced pseudovirus infection for the two GPΔmuc trimer groups, the FR group, and the two 60-meric E2p/I3-01v9 groups are observed at w2, w5, and w8, respectively, and are highlighted in dashed rectangles. Source data are provided as a Source Data file.

a

GPΔmuc-WL^{2}P^2-1TD0-Avi-Biot

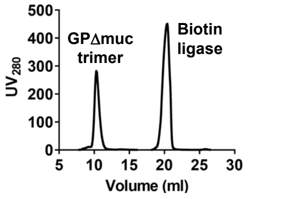

b

Gating strategies in bulk B cell sorting

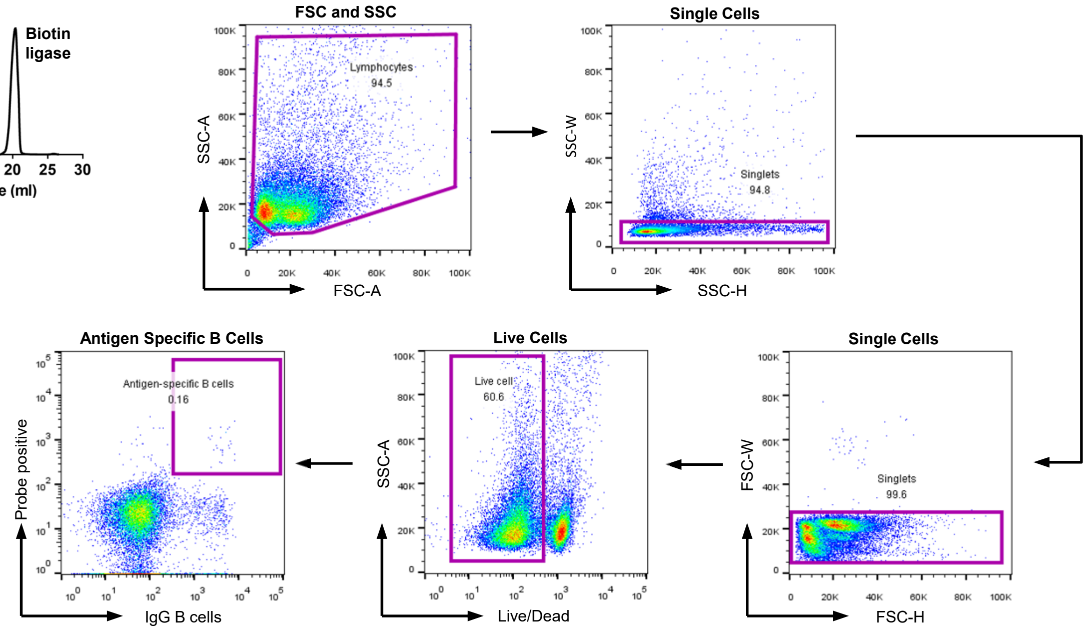

EBOV GPΔmuc-specific sorting of mouse splenic B cells from five vaccine groups

GPΔmuc-Foldon			GPΔmuc-WL^{2}P^2-Foldon			GPΔmuc-WL^{2}P^2-5GS-FR			GPΔmuc-WL^{2}P^2-E2p-L4P			GPΔmuc-WL^{2}P^2-10GS-I3-01v9-L7P		
Mouse	Sorted B cells	%Splenic B cells	Mouse	Sorted B cells	%Splenic B cells	Mouse	Sorted B cells	%Splenic B cells	Mouse	Sorted B cells	%Splenic B cells	Mouse	Sorted B cells	%Splenic B cells
G5-1	760	0.03579	G7-1	1134	0.07849	G3-1	1856	0.07481	G4-1	1360	0.07285	G10-2	401	0.07874
G5-3	582	0.04646	G7-3	585	0.18874	G3-2	1146	0.04230	G4-2	906	0.09027	G10-3	602	0.10956
G5-6	857	0.04580	G7-4	1043	0.18983	G3-5	1362	0.04844	G4-3	1369	0.08957	G10-4	443	0.06507
G5-7	1065	0.04149	G7-6	949	0.19321	G3-7	2864	0.10775	G4-6	1400	0.08291	G10-5	871	0.04330
G5-8	752	0.04904	G7-8	982	0.09138	G3-8	843	0.06591	G4-7	1485	0.05536	G10-8	372	0.05807

c

Next-generation sequencing (NGS) data obtained for EBOV GP-specific mouse splenic B cells [a]

Vaccine antigen	Mouse	Chain	N_{Chain}	N_{Step5}	<Length>	N_{Usable}
GPΔmuc-foldon	G5-1	H [b]	241,888	152,926	369.7	83,074
		K	320,999	271421	335.3	244,765
	G5-3	H [b]	151,041	38,965	365.7	25,901
		K	254,461	190,810	332.5	159,340
	G5-6	H	164,049	72,966	364.3	54,123
		K	149,922	122,358	328.8	112,897
	G5-7	H [b]	127,583	31,333	362.8	21,695
		K	132,822	101,243	331.6	86,936
	G5-8	H [b]	416,364	146,889	363.5	98,999
		K	319,964	245,757	331.5	219,409
GPΔmuc-WL^{2}P^2-foldon	G7-1	H [b]	372,642	174,424	363.5	116,260
		K	147,494	121,251	330.8	107536
	G7-3	H [b]	225,662	109,976	363.4	75,064
		K	207,512	158,440	332.6	142,203
	G7-4	H [b]	195,714	58,996	364.4	39,423
		K	181,907	143,388	333.8	126,382
	G7-6	H [b]	203,093	49,877	361.0	34,366
		K	234,578	193,315	334.7	174,967
	G7-8	H [b]	193,745	96,042	352.4	75,369
		K	166,563	138,767	334.7	118,193
GPΔmuc-WL^{2}P^2-5GS-FR [c]	G3-1	H [b]	181,414	15,426	361.6	9,391
		K	172,971	110,166	333.9	99,231
	G3-2	H [b]	196,037	24,375	366.4	15,433
		K	165,297	118,826	332.0	106,667
	G3-5	H [b]	171,116	13,134	367.4	8,154
		K	202,059	149,466	335.5	133,378
	G3-7	H [b]	149,991	10,289	365.4	6,028
		K	192,304	132,601	329.7	120,184
	G3-8	H	250,631	171,124	362.3	132,011
		K	234,322	162,071	328.2	139,171

Vaccine antigen	Mouse	Chain	N_{Chain}	N_{Usable}	<Length>	$Perc_{Usable}$
GPΔmuc-WL^{2}P^2-E2p-L4P	G4-1	H	132,044	55,538	362.8	42,292
		K	173,895	138,355	331.8	118,788
	G4-2	H	95,696	71,107	360.5	57,045
		K	155,727	132,125	335.0	117,275
	G4-3	H	105,559	85,392	362.1	72,646
		K	75,330	59,105	332.3	48,150
	G4-6	H	139,269	106,616	365.5	84,238
		K	146,092	113,590	335.2	105,666
	G4-7	H [b, d]	672,503	542,016	365.2	369,668
		K	519,784	405,118	333.6	362,987
GPΔmuc-WL^{2}P^2-10GS-I3-01v9-L7P	G10-2	H	452,882	268,275	365.5	212,617
		K	290,177	258,708	328.5	233,447
	G10-3	H	470,021	278,422	362.0	210,314
		K	223,350	207,358	333.2	191,472
	G10-4	H	297,056	261,706	363.5	215,196
		K	226,568	203,176	336.7	187,416
	G10-5	H	190,727	138,889	366.4	106,419
		K	237,420	197,478	331.3	183,103
	G10-8	H	260,502	90,086	364.6	72,427
		K	350,497	270,707	334.3	229,614

[a] Listed items include the vaccine antigen, mouse sample ID, number of V_H/V_K chains prior to the antibodyomics pipeline processing, number of V_H/V_K chains at step 5 of the pipeline processing, average read length, and number of usable chains after removing fragments with a V-gene alignment of 250bp or shorter.

[b] Due to the difficulty in library preparation, a smaller heavy chain dataset was obtained for a number of mouse samples, especially those from the trimer and FR groups. Two additional Ion S5 sequencing runs using the Ion 530 chip was performed to obtain more heavy chain reads to facilitate the profile analysis.

[c] G3-1, 5, and 7 showed especially low read-out and the profiles were only used for qualitative comparison with other vaccine groups but not for statistical analysis.

[d] Due to the large size of the dataset obtained from the additional NGS run, pre-filtering with a read length cutoff of 400bp was performed before merging it with the heavy chain dataset obtained from the initial run.

Fig. S9

d GPΔmuc-foldon group

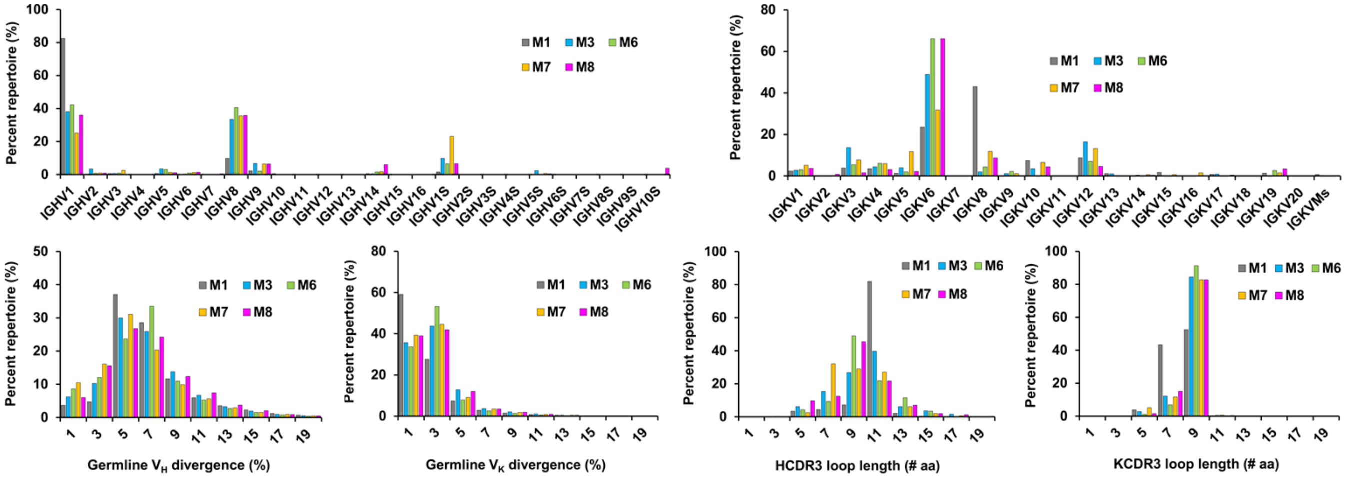

e GPΔmuc-WL²P²-5GS-FR group

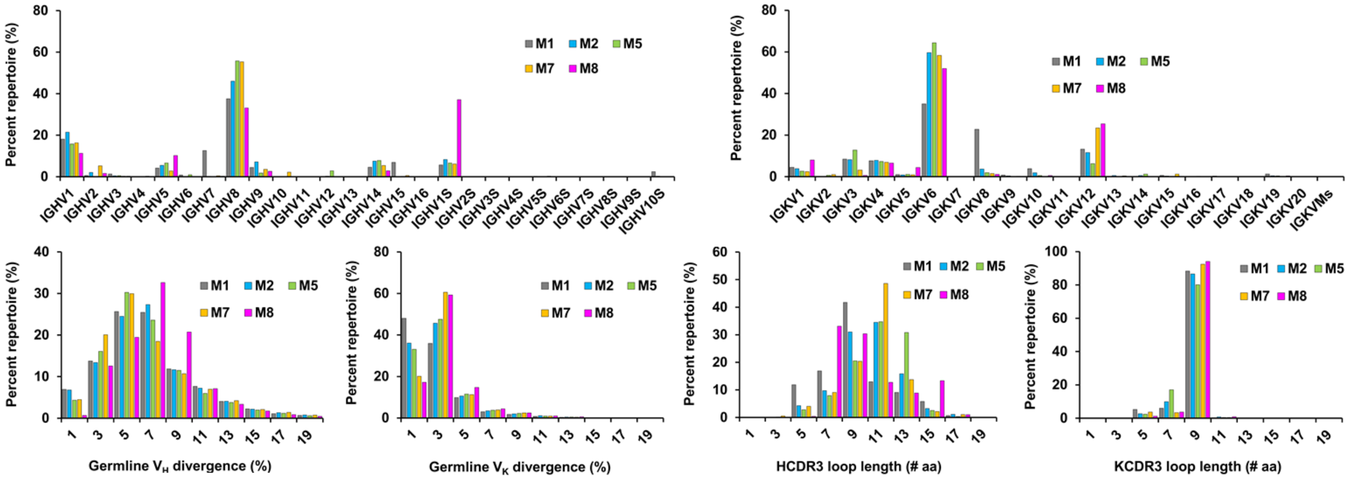

f GPΔmuc-WL²P²-10GS-I3-01v9-L7P group

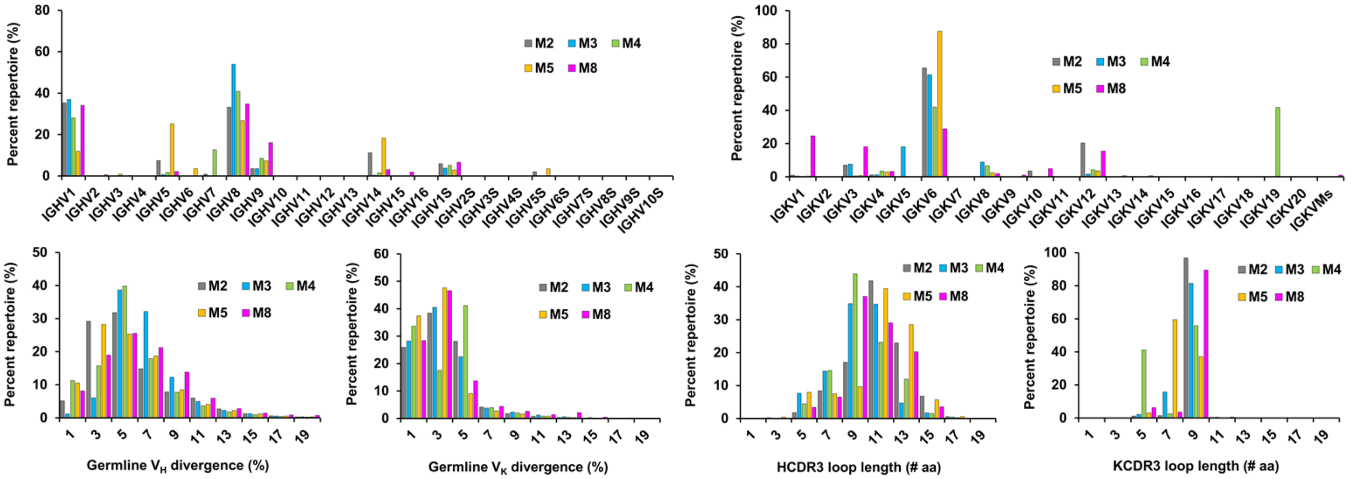

Fig. S9. Unbiased repertoire analysis of bulk-sorted EBOV GP-specific mouse splenic B cells. (**a**) SEC profile of a biotinylated Avi-tagged EBOV GPΔmuc trimer, termed GPΔmuc-WL^{2}P^2-1TD0-Avi-Biot, obtained from a HiLoad Superdex 200 16/600 column. 1TD0 is a trimerization domain used to stabilize GPΔmuc and to mask foldon-specific B cells in the two trimer vaccine groups. (**b**) Top: gating strategies used in the antigen-specific mouse B cell sorting (Step 1: remove cell debris; Steps 2 and 3: exclude clumped or sticky cells to ensure that only single cells remain; Step 4: remove dead cells; Step 5: identify EBOV GP-specific B cells). Bottom: Summary of EBOV GPΔmuc-specific bulk sorting of mouse splenic B cells from five vaccine groups. (**c**) Antibodyomics analysis of repertoire NGS data obtained for GPΔmuc-specific mouse splenic B cells. Distribution of critical B-cell properties are shown for three vaccine groups, in which mice were immunized with (**d**) GPΔmuc-foldon, (**e**) GPΔmuc-WL^{2}P^2-5GS-FR, and (**f**) GPΔmuc-WL^{2}P^2-10GS-I3-01v9-L7P. For each group, distributions are shown for germline VH/VK gene usage (top), germline VH/VK divergence (bottom left) and CDRH/K3 loop length (bottom right). Source data are provided as a Source Data file.

Table S1. Primers used in EBOV GP construct design, nanoparticle construct design, and next-generation sequencing analysis of mouse splenic B cells.

a. Primers used in EBOV GP construct design

Leader-SalI-Forward	ACCGTCGTCGACGCCACCATGGGTGTCACCGGGATACTCCAG
GPΔmuc/GP_{ECTO}-NheI-BamHI-Reverse	ATGATGGGATCCCTATTAGCTAGCATCTACAAAGTCGTGGATGATCTGGTC
GPΔmuc-2WPZ-NheI-BamHI-Reverse	ATGATGGGATCCCTATTAGCTAGCTCGTTCGCCGACAAGTTTCTTGAG
GPΔmuc-L-NheI-BamHI-Reverse	ATGATGGGATCCCTATTAGCTAGCATCGGGCAATGTTTTATCTACAAA
GPΔmuc-Ext-NheI-BamHI-Reverse	ATGATGGGATCCCTATTAGCTAGCGTTATCGTTGTCTCCCTGATCGGGCAATGT
W615L-Forward	TGTTGCATAGAGCCGCACGATTTTACCAAGAATATCACCGATAAA
W615L-Reverse	TTTATCGGTGATATTCTTGGTGAGATCGTGCGGCTCTATGCAACA
GPΔmuc-P1-Forward	CTCCAGCTCTTCCTCAGGGCCCCTACGGAACTGCGAACGTTTAGC
GPΔmuc-P1-Reverse	GCTAAACGTTCGCAGTTCCGTAGGGGCCCTGAGGAAGAGCTGGAG
GPΔmuc-P2-Forward	CAGCTCTTCCTCAGGGCCACTCCGGAACTGCGAACGTTTAGCATT
GPΔmuc-P2-Reverse	AATGCTAAACGTTCGCAGTTCCGGAGTGGCCCTGAGGAAGAGCTG
GPΔmuc-P3-Forward	CAGCTCTTCCTCAGGGCCACTACGCCACTGCGAACGTTTAGCATTCTTAAT
GPΔmuc-P3-Reverse	ATTAAGAATGCTAAACGTTCGCAGTGGCGTAGTGGCCCTGAGGAAGAGCTG
GPΔmuc-P4-Forward	CTCTTCCTCAGGGCCACTACGGAACCGCGAACGTTTAGCATTCTTAATAGG
GPΔmuc-P4-Reverse	CCTATTAAGAATGCTAAACGTTCGCGGTTCCGTAGTGGCCCTGAGGAAGAG
GPΔmuc-P5-Forward	TTCCTCAGGGCCACTACGGAACTGCCAACGTTTAGCATTCTTAATAGGAAA
GPΔmuc-P5-Reverse	TTTCCTATTAAGAATGCTAAACGTTGGCAGTTCCGTAGTGGCCCTGAGGAA
GPΔmuc-P6-Forward	CTCAGGGCCACTACGGAACTGCGACCGTTTAGCATTCTTAATAGGAAAGCG
GPΔmuc-P6-Reverse	CGCTTTCCTATTAAGAATGCTAAACGGTCGCAGTTCCGTAGTGGCCCTGAG
GPΔmuc-P7-Forward	AGGGCCACTACGGAACTGCGAACGCCTAGCATTCTTAATAGGAAAGCGATA
GPΔmuc-P7-Reverse	TATCGCTTTCCTATTAAGAATGCTAGGCGTTCGCAGTTCCGTAGTGGCCCT
GPΔmuc-P8-Forward	GCCACTACGGAACTGCGAACGTTTCCCATTCTTAATAGGAAAGCGATAGAT
GPΔmuc-P8-Reverse	ATCTATCGCTTTCCTATTAAGAATGGGAAACGTTCGCAGTTCCGTAGTGGC
GPΔmuc-SS1a-Q5-Forward	TGCGCGGGTGATTTTGCTTGTCATAAAGAAGGCGCGTTTTTC
GPΔmuc-SS1a-Q5-Reverse	TGGTCCTGTCCCTGACACCTTGTG
GPΔmuc-SS1b-Q5-Forward	GGTCTTGCTTGGATACCCTGCTTCGGACCAGCCGCTGAAGGA
GPΔmuc-SS1b-Q5-Reverse	TATCGCAGCCCCTTCGTCTTGTGT
GPΔmuc-SS2a-Q5-Forward	AGGTGGGGTTTTAGGTCCTGTGTTCCCCCTAAGGTTGTTAAT
GPΔmuc-SS2a-Q5-Reverse	TTTGGTTGCTGATGGGACGTCAGT
GPΔmuc-SS2b-Q5-Forward	ATACCCTACTTCGGACCATGCGCTGAAGGAATCTACATA
GPΔmuc-SS2b-Q5-Reverse	CCAAGCAAGACCTATCGCAGCCCC

GPΔmuc-SS3a-Q5-Forward	AACTTGCATTATTGGACCTGTCAAGACGAAGGGGCTGCGATA
GPΔmuc-SS3a-Q5-Reverse	AGGGTTACATTTTGGCTGGGCATT
GPΔmuc-SS3b-Q5-Forward	CTCCAGCTCTTCCTCAGGTGCACTACGGAACTGCGAACGTTT
GPΔmuc-SS3b-Q5-Reverse	GGCTTGCGTGGTCTCATTGGCAAG
GPΔmuc-SS4a-Q5-Forward	TGCGCGGGTGATTTTGCTTTTCATAAAGAATGCGCGTTTTTCCTCTATG ACCGC
GPΔmuc-SS4a-Q5-Reverse	TGGTCCTGTCCCTGACACCTTGTG
GPΔmuc-SS4b-Q5-Forward	GCGATAGGTCTTGCTTGGTGTCCCTACTTCGGACCAGCCGCT
GPΔmuc-SS4b-Q5-Reverse	AGCCCCTTCGTCTTGTGTGGTCCA
GPΔmuc-SS5a-Q5-Forward	AACTTGCATTATTGGACCACACAATGCGAAGGGGCTGCGATAGGTCTT
GPΔmuc-SS5a-Q5-Reverse	AGGGTTACATTTTGGCTGGGCATT
GPΔmuc-SS5b-Q5-Forward	CTCCAGCTCTTCCTCAGGTGCACTACGGAACTGCGAACGTTT
GPΔmuc-SS5b-Q5-Reverse	GGCTTGCGTGGTCTCATTGGCAAG
GPΔmuc-SS6a-Q5-Forward	CAAGTTTCAGATGTGGACTGTCTTGTCTGTCGCGATAAACTT
GPΔmuc-SS6a-Q5-Reverse	AAGCGCGGAGTTGTGGATCACACC
GPΔmuc-SS6b-Q5-Forward	CTCCTCCAAAGGTGGGGCTGTACGTGTCATATATTGGGTCCC
GPΔmuc-SS6b-Q5-Reverse	GAAATCTATCGCTTTCCTATTAAG

b. Primers used in EBOV GP-presenting nanoparticle construct design

Ferritin-BamHI-Reverse	ATGATGGGATCCCTATTATGATTTGCGGCTCTTTGCGATACC
E2p-BamHI-Reverse	ATGATGGGATCCCTATTACATCAGAAGCAGCTCGGGGTCACT
I3-01-BamHI-Reverse	ATGATGGGATCCCTATTACTCAGTACATCCCCGTATTTT
LD1-BamHI-Reverse	ATGATGGGATCCCTATTACCGTCGCTCATTAACAACCACTTC
LD2-BamHI-Reverse	ATGATGGGATCCCTATTAGTGAGAGTCGAGGGCAATCTGCAA
LD3-BamHI-Reverse	ATGATGGGATCCCTATTACCGTTCCCTGTGTGGTGCGACTTC
LD4-BamHI-Reverse	ATGATGGGATCCCTATTACTTTTCAAACTGTGGGTGTGACCA
LD5-BamHI-Reverse	ATGATGGGATCCCTATTACCACAAGCACCCTTTGCATGCTTC
LD6-BamHI-Reverse	ATGATGGGATCCCTATTACTTATTCGTATACATTTCGAGGTA
LD7-BamHI-Reverse	ATGATGGGATCCCTATTAGTCTGCCCTGCGTGAGTTCCAGCG
LD8-BamHI-Reverse	ATGATGGGATCCCTATTATCGCCGTGCTTCGCGCAGTCGAGT
LD9-BamHI-Reverse	ATGATGGGATCCCTATTACTCATTTATGCTGCTCTGAAGTGC
PADRE-BamHI-Reverse	ATGATGGGATCCCTATTAGGCGGCGGCCTTCAGGGTCCAGGCGGC

c. Primers used in preparation of antibody chain libraries from mouse splenic B cells. [a]

5'-RACE-P1-adaptor	CCACTACGCCTCCGCTTTCCTCTCTATGGGCAGTCGGTGATAAGCAGT GGTATCAACGCAGAGTAC
3'-Cγ1-inner-A-adaptor [b]	CCATCTCATCCCTGCGTGTCTCCGACTCAG [barcode] GCTCAGGGAAATAGCCCTTGAC
3'-Cγ2c-inner-A-adaptor [b]	CCATCTCATCCCTGCGTGTCTCCGACTCAG [barcode] GCTCAGGGAAATAACCCTTGAC
3'-Cγ2b-inner-A-adaptor [b]	CCATCTCATCCCTGCGTGTCTCCGACTCAG [barcode] ACTCAGGGAAGTAGCCCTTGAC
3'-Cγ3-inner-A-adaptor [b]	CCATCTCATCCCTGCGTGTCTCCGACTCAG [barcode] GCTCAGGGAAGTAGCCTTTGAC
3'-Cμ-inner-A-adaptor [b]	CCATCTCATCCCTGCGTGTCTCCGACTCAG [barcode] AGGGGGAAGACATTTGGGAAGGAC
3'-mCκ-outer-A-adaptor [b]	CCATCTCATCCCTGCGTGTCTCCGACTCAG [barcode] GATGGTGGGAAGATGGATACAGTT

[a] The 3' primers are adapted from Tiller et al., *Journal of Immunological Methods* 350, 183-193 (2009).
[b] Ion Xpress Barcodes 1-20 [in blue] are used to differentiate antibody libraries from different mice.

Table S2. Data collection and refinement statistics for EBOV GPΔmuc trimers.

**Data Collection**	**GPΔmuc-WL^2-foldon**	**GPΔmuc-WL^{2}P^2-foldon**
Beamline	APS 23-ID-D	SSRL 12-2
Wavelength (Å)	1.0332	0.97946
Resolution (Å)[a]	49.00-2.28 (2.34-2.28)	49.44-3.20 (3.26-3.20)
Space group	R32	P321
Unit cell (Å)	114.58, 114.58, 312.38	114.06, 114.06, 136.22
(°)	90 90 120	90 90 120
Total reflections	156,593	153,527
Unique reflections	34,904 (1725)	17,343 (864)
Multiplicity	4.5 (3.3)	8.9 (8.7)
Completeness (%)	96.4 (97.3)	99.9 (99.8)
Mean (I)/ σ_I	8.0 (0.9)	8.4 (0.45)
R_{merge}[b](%)	15.0(124)	29.9 (524)
R_{meas}[c] (%)	16.8 (147)	31.9 (557)
R_{pim}[d] (%)	7.4 (77.5)	10.8 (187)
$CC_{1/2}$[e] (%)	78.1 (32.5)	77.6 (31.3)
**Refinement**		
Refinement resolution (Å)[a]	49.00-2.28 (2.35-2.28)	49.44-3.20 (3.28-3.20)
# reflections in refinement (work/free)	33177/1715	16245/837
R_{work} (%)	20.0 (33.8)	27.7 (36.0)
R_{free} (%)	23.7 (36.6)	32.2 (37.1)
# Protein atoms	2942	3123
# Carbohydrate atoms	154	139
# Waters	138	0
# Protein residues	374	398
Bond r.m.s. deviation (Å)	0.002	0.013
Angle r.m.s. deviation (º)	0.53	1.80
Ramachandran favored, allowed, outliers (%)(*83*)	96.9, 3.1, 0.0	90.3, 9.4, 0.3
Clashscore[f]	3.0	9.0
Wilson B (Å^2)	43	83
Average B (Å^2)	58	94
Protein	57	94
PDB ID	7JPI	7JPH

[a]Numbers in parentheses are for highest resolution shell

[b]$R_{merge} = \Sigma_{hkl} \Sigma_{i=1,n}|I_i(hkl) – <I(hkl)>| / \Sigma_{hkl} \Sigma_{i=1,n} I_i(hkl)$

[c]$R_{meas} = \Sigma_{hkl} \sqrt{(n/n-1)} \Sigma_{i=1,n}|I_i(hkl) – <I(hkl)>| / \Sigma_{hkl} \Sigma_{i=1,n} I_i(hkl)$

[d]$R_{pim} = \Sigma_{hkl} \sqrt{(1/n-1)} \Sigma_{i=1,n}|I_i(hkl) – <I(hkl)>| / \Sigma_{hkl} \Sigma_{i=1,n} I_i(hkl)$

[e]$CC_{1/2}$ = Pearson Correlation Coefficient between two random half datasets

[f] Number of unfavorable all-atom steric overlaps ≥ 0.4Å per 1000 atoms
