# Supplementary material for: Single-component multilayered self-assembling nanoparticles presenting rationally designed glycoprotein trimers as Ebola virus vaccines
Source: Nat Commun. 2021 May 11;12:2633. doi: 10.1038/s41467-021-22867-w (PMC8113551; doi:10.1038/s41467-021-22867-w)
Supplement: Supplementary file 3 — Reporting Summary [file 41467_2021_22867_MOESM3_ESM.pdf]

nature research

Corresponding author(s): Ian A. Wilson and Jiang Zhu

Last updated by author(s): Apr 2, 2021

Reporting Summary

Nature Research wishes to improve the reproducibility of the work that we publish. This form provides structure for consistency and transparency in reporting. For further information on Nature Research policies, see our Editorial Policies and the Editorial Policy Checklist.

Statistics

For all statistical analyses, confirm that the following items are present in the figure legend, table legend, main text, or Methods section.

n/a	Confirmed	
☐	☒	The exact sample size (*n*) for each experimental group/condition, given as a discrete number and unit of measurement
☐	☒	A statement on whether measurements were taken from distinct samples or whether the same sample was measured repeatedly
☐	☒	The statistical test(s) used AND whether they are one- or two-sided<br>*Only common tests should be described solely by name; describe more complex techniques in the Methods section.*
☐	☒	A description of all covariates tested
☐	☒	A description of any assumptions or corrections, such as tests of normality and adjustment for multiple comparisons
☐	☒	A full description of the statistical parameters including central tendency (e.g. means) or other basic estimates (e.g. regression coefficient) AND variation (e.g. standard deviation) or associated estimates of uncertainty (e.g. confidence intervals)
☐	☒	For null hypothesis testing, the test statistic (e.g. *F*, *t*, *r*) with confidence intervals, effect sizes, degrees of freedom and *P* value noted<br>*Give P values as exact values whenever suitable.*
☒	☐	For Bayesian analysis, information on the choice of priors and Markov chain Monte Carlo settings
☒	☐	For hierarchical and complex designs, identification of the appropriate level for tests and full reporting of outcomes
☒	☐	Estimates of effect sizes (e.g. Cohen's *d*, Pearson's *r*), indicating how they were calculated

Our web collection on statistics for biologists contains articles on many of the points above.

Software and code

Policy information about availability of computer code

Data collection	SEC data were collected using the Unicorn 7.5 software (GE Healthcare). BN-PAGE images were collected using the Image Lab v6.0 software. BLI (Octet) data were collected by the Data aquisition 8.2 (FORTEBIO). DSC data were collected by the MicroCal PEAQ-DSC software v1.52 (Malvern Panalytical). ELISA data were collected using the PerkinElmer 2030 v4.0 software (PerkinElmer). Neutralization data were collected using the Gen 5 3.05 software (BioTek). Flow cytometry data were collected using the Summit 6.3 software.
Data analysis	Sequence logo analysis was done and the image was generated using the WebLoGo v2.8 software. DSC data were analyzed using the Origin 7.0 software. GraphPad Prism 8.4.3 was used for the analysis of ELISA and neutralization data. BLI (Octet) data were analyzed by Data analysis 8.2 (FORTEBIO). Antibody next-generation sequencing data were analyzed using Antibodyomics v1.0. Phaser v2.8.3 from the CCP4i suite v7.1.002, MOLREP v11.7.02, Coot v0.8.9, Refmac v5.8.0258, MolProbity v4.1, Leginon v3.0, cryoSPARC v2.2, PyMOL v2.3.4, and Chimera v1.13 were used for structural analysis.

For manuscripts utilizing custom algorithms or software that are central to the research but not yet described in published literature, software must be made available to editors and reviewers. We strongly encourage code deposition in a community repository (e.g. GitHub). See the Nature Research guidelines for submitting code & software for further information.

Data

Policy information about availability of data

All manuscripts must include a data availability statement. This statement should provide the following information, where applicable:

- Accession codes, unique identifiers, or web links for publicly available datasets
- A list of figures that have associated raw data
- A description of any restrictions on data availability

A detailed data availability statement is provided: The X-ray crystallographic coordinates for two rationally redesigned GPmuc structures in this study have been

deposited in the Protein Data Bank (PDB, https://www.rcsb.org/), under accession codes 7JPI (http://doi.org/10.2210/pdb7jpi/pdb) and 7JPH (http://doi.org/10.2210/pdb7jph/pdb). The mouse B-cell NGS datasets have been deposited in the NIH Sequence Read Archive (SRA, https://www.ncbi.nlm.nih.gov/sra), with the identifier PRJNA718964 (https://www.ncbi.nlm.nih.gov/bioproject/PRJNA718964/). The authors declare that the data supporting the findings of this study are available within the article and its Supplementary Information files. Source data are provided with this paper.

Field-specific reporting

Please select the one below that is the best fit for your research. If you are not sure, read the appropriate sections before making your selection.

- [x] Life sciences
- [ ] Behavioural & social sciences
- [ ] Ecological, evolutionary & environmental sciences

For a reference copy of the document with all sections, see nature.com/documents/nr-reporting-summary-flat.pdf

Life sciences study design

All studies must disclose on these points even when the disclosure is negative.

Sample size	No statistical analysis was performed to predetermine the group size. The numbers used in the current study are rather standard for the field and have been used in our previous studies (e.g. groups of 4-10 mice and 4 rabbits used in He et al., Sci Adv, 4:eaau6769, 2018; He et al., Sci Adv, 6:eaaz6225, 2020). Here, groups of 8 mice were used to evaluate a large set of EBOV GP and nanoparticles, and groups of 4 rabbits were immunized to further validate the immunogenicity for a subset of EBOV GP and nanoparticles.
Data exclusions	Two mice were excluded from the longitudinal serum ELISA analyses following their death (the previous time points still included). They were also removed from the neutralization analysis of mouse IgG purified from the last time point, week 11.
Replication	In both mouse and rabbit studies, each animal was immunized 4 times. Antibody binding to GP and GP nanoparticle was confirmed using two different methods: ELISA and BLI (Octet). All plasma binding assays were performed with duplicates. Due to the limited sample availability, neutralization assays using purified mouse IgG were performed once without duplicates. As sufficient plasma samples can be collected from rabbits, neutralization assays using purified rabbit IgG were performed with duplicates.
Randomization	No randomization was needed, because the animals in this study were homogeneous in sex, age, and weight. Six-to-eight-week-old female BALB/c wild type mice were purchased from The Jackson Laboratory. Three-to-four-month-old female New Zealand White rabbits of 2.5-4.0kg were purchased by ProSci from the Western Oregon Rabbit Co.
Blinding	The animals in this study (mice and rabbits) were homogeneous in sex, age, and weight. Blinding is not relevant in this study. Animal samples were collected and analyzed using standardized protocols and assays with proper controls. There is no subjective assessment of the animals and the collected animal samples.

Reporting for specific materials, systems and methods

We require information from authors about some types of materials, experimental systems and methods used in many studies. Here, indicate whether each material, system or method listed is relevant to your study. If you are not sure if a list item applies to your research, read the appropriate section before selecting a response.

Materials & experimental systems

n/a	Involved in the study	
☐	☒	Antibodies
☐	☒	Eukaryotic cell lines
☒	☐	Palaeontology and archaeology
☐	☒	Animals and other organisms
☒	☐	Human research participants
☒	☐	Clinical data
☒	☐	Dual use research of concern

Methods

n/a	Involved in the study	
☒	☐	ChIP-seq
☐	☒	Flow cytometry
☒	☐	MRI-based neuroimaging

Antibodies

Antibodies used	Anti-human IgG (Jackson ImmunoResearch #109-035-008), anti-mouse IgG (Jackson ImmunoResearch #115-036-008), and rabbit IgG (Jackson ImmunoResearch #111-035-144) conjugated to horseradish peroxidase (HRP) were used in ELISA binding assays.<br><br>Mouse (BD Biosciences #553142) Fc Block and anti-Mouse IgG FITC (Jackson ImmunoResearch #115-095-071) were used for cytometric analysis of GP-specific mouse splenic B cells.<br><br>All antibodies used in ELISA and BLI assays and for packing antibody columns were synthesized and then produced in-house in ExpiCHO cells. These antibodies include KZ52 (from PDB 3CSY), mAb114 (from PDB 5FHC), mAb100 (from PDB 5FHC), c2G4 (from PDB 5KEL), c4G7 (from PDB 5KEN), c13C6 (from PDB 5KEL), CA45 (from PDB 6EAY), ADI-15878 (from PDB 6DZN), ADI-15946 (from PDB 6MAM), and BDBV223 (from PDB 6N7U).
Validation	All the commercially available antibodies have been validated by their manufacturers, BD Biosciences and Jackson ImmunoResearch. Validation reports can be found on their websites using the catalogue number indicated above (also see below). The flow cytometry analysis performed in this study also validated the use of these antibodies.<br><br>https://www.jacksonimmuno.com/catalog/products/109-035-008<br>https://www.jacksonimmuno.com/catalog/products/115-036-008<br>https://www.bdbiosciences.com/us/applications/research/b-cell-research/surface-markers/mouse/purified-rat-anti-mouse-cd16cd32-mouse-bd-fc-block-24g2/p/553142<br>https://www.jacksonimmuno.com/catalog/products/115-095-071<br><br>The amino acid sequences of known EBOV antibodies were obtained from the Protein Data Bank (PDB) for synthesis and production. The ELISA and BLI binding assays and the neutralization assays performed in this study validated the function of these antibodies.

Eukaryotic cell lines

Policy information about cell lines

Cell line source(s)	HEK293 F cells (Thermo Fisher)<br>HEK293 T cells (ATTC)<br>ExpiCHO cells (Thermo Fisher)<br>TZM-bl cells (NIH AIDS Reagent program)
Authentication	These commonly used cell lines were obtained from and authenticated by vendors and government agencies
Mycoplasma contamination	The cell lines were not contaminated by mycoplasma as determined by using the Lonza Mycoplasma Detection Kit.
Commonly misidentified lines (See ICLAC register)	None

Animals and other organisms

Policy information about studies involving animals; ARRIVE guidelines recommended for reporting animal research

Laboratory animals	Six-to-eight-week-old female BALB/c wild type mice were from The Jackson Laboratory were used for immunization.<br>Three-to-four-month-old female New Zealand White rabbits of 2.5-4.0kg were purchased from the Western Oregon Rabbit Co. for immunization.
Wild animals	This study did not use wild animals
Field-collected samples	This study did not involve samples collected from the field.
Ethics oversight	The mouse immunization was performed following the IACUC protocol approved by The Scripps Research Institute.<br>The rabbit immunization was performed by ProSci under ProSci's IACUC protocol APF-1A and related amendments, which were originally approved on 10/01/2019 and are set to expire on 10/01/2021.

Note that full information on the approval of the study protocol must also be provided in the manuscript.

Flow Cytometry

Plots

Confirm that:

☒ The axis labels state the marker and fluorochrome used (e.g. CD4-FITC).

☒ The axis scales are clearly visible. Include numbers along axes only for bottom left plot of group (a 'group' is an analysis of identical markers).

☒ All plots are contour plots with outliers or pseudocolor plots.

☒ A numerical value for number of cells or percentage (with statistics) is provided.

Methodology

Sample preparation	Spleens were harvested from immunized mice 15 days after the last immunization and cell suspension was prepared.
Instrument	MoFlo Astrios (Beckman Coulter)
Software	Summit 6.3
Cell population abundance	GPdmuc-specific mouse splenic B cells after sorting showed frequencies between 0.03579% and 0.19321%.
Gating strategy	Antigen-specific mouse splenic B cells were isolated by gating on single cells, Live/dead marker negative (Fixable Aqua Dead Cell Stain kit, Thermo Fisher L34957), mouse IgG positive (FITC, Jackson ImmunoResearch 115-095-071), and biotinylated EBOV GP positive (APC, Thermo Fisher SA1005).

☒ Tick this box to confirm that a figure exemplifying the gating strategy is provided in the Supplementary Information.
