# Supplementary material for: Single-component multilayered self-assembling nanoparticles presenting rationally designed glycoprotein trimers as Ebola virus vaccines
Source: Nat Commun. 2021 May 11;12:2633. doi: 10.1038/s41467-021-22867-w (PMC8113551; doi:10.1038/s41467-021-22867-w)
Supplement: Supplementary file 2 — Peer Review File [file 41467_2021_22867_MOESM2_ESM.pdf]

Editorial Note: This manuscript has been previously reviewed at another journal that is not operating a transparent peer review scheme. This document only contains reviewer comments and rebuttal letters for versions considered at Nature Communications.

Reviewers' Comments:

Reviewer #1:
Remarks to the Author:
The authors have improved the manuscript by addressing the technical concerns of the initial reviews.
